# Development of a lytic Ralstonia phage cocktail and evaluation of its control efficacy against tobacco bacterial wilt

**DOI:** 10.3389/fpls.2025.1554992

**Published:** 2025-03-13

**Authors:** Haoxin He, Ke Yi, Lei Yang, Yongfeng Jing, Lifu Kang, Zhihao Gao, Dong Xiang, Ge Tan, Yunsheng Wang, Qian Liu, Lin Xie, Shiya Jiang, Tianbo Liu, Wu Chen

**Affiliations:** ^1^ College of Plant Protection, Hunan Agricultural University, Changsha, China; ^2^ Tobacco Leaf Raw Material Procurement Center, China Tobacco Hunan Industrial Co., Ltd, Changsha, China; ^3^ Plant Protection Research Center, Hunan Tobacco Science Research Institute, Changsha, China

**Keywords:** bacteria wilt (BW), *Ralstonia solanacearum*, Ralstonia phage, phage cocktail, control efficacy, tail fiber

## Abstract

**Introduction:**

Bacterial wilt (BW) caused by *Ralstonia pseudosolanacearum* is a devastating soil-borne disease. Bacteriophages are important biocontrol resources that rapidly and specifically lyse host bacteria, showing good application potential in agricultural production.

**Methods:**

This study isolated nine phages (YL1–YL9) and, using host range and pot experiments, identified two broader host range phages (YL1 and YL4) and two higher control efficacy phages (YL2 and YL3), which were combined to obtain five cocktails (BPC-1–BPC-5).

**Results:**

Pot experiments showed that BPC-1 (YL3 and YL4) had the highest control efficacy (99.25%). Biological characterization revealed that these four phages had substantial thermal stability and pH tolerance. Whole genome sequencing and analysis showed that YL1, YL2, YL3, and YL4 belonged to the genus *Gervaisevirus*. AlphaFold 3 predictions of tail fiber protein II structures showed that YL1 differed significantly from the other phages. Amino acid sequence alignment revealed that the ORF66 (YL1) “tip domain” of contained a higher proportion of aromatic and positively charged amino acids. However, the surface of the ORF69 (YL4) “tip domain” exhibited more positively charged residues than ORF66 (YL2) and ORF70 (YL3). These characteristics are hypothesized to confer a broader host range to YL1 and YL4.

**Discussion:**

This study demonstrates that phages assembling a broad host range and high control efficacy have better biocontrol potential, providing high-quality resources for the biological control of BW.

## 1 Introduction

The *Ralstonia solanacearum* species complex (RSSC) infects over 200 plant species from 50 families, including tobacco, tomato, potato, and pepper, causing typical bacterial wilt (BW) (Denny, 2000; Lowe-Power et al., 2020; Paudel et al., 2020). Surveys have shown that BW is the second most frequent plant disease globally, causing annual economic losses of about USD 1 billion (Mansfield et al., 2012; Elphinstone, 2005). RSSC has high variability and complex genetic diversity (Jiang et al., 2017). Based on its geographical origins and phylogenetic analysis, RSSC can be divided into three species: *R. pseudosolanacearum* (formerly Asian phylotype I and African phylotype III), *R. solanacearum* (formerly American phylotype II), and *R. syzygii* (formerly the Indonesian phylotype) (Paudel et al., 2020; Zhao et al., 2023).

Lytic Ralstonia phages that infect hosts have the following characteristics: fast infection, short lysis time, and high host specificity (Dion et al., 2020; Mushegian, 2020). They reduce the number of host bacteria in the environment in a short time, without causing harm to beneficial microorganisms in the environment, while simultaneously regulating rhizosphere microbial composition and function to collectively resist pathogen invasion. (Trivedi et al., 2020; Ji et al., 2021; Markwitz et al., 2022). Therefore, phage therapy is considered an effective method for BW control (Buttimer et al., 2017). Askora et al. (2017) isolated and purified Ralstonia phage ϕRSY1 from the soil, and root irrigation and stem injection with *R. solanacearum* M4S infected with ϕRSY1 (10^8^ cell/mL) significantly reduced the incidence and disease index of tomato BW. Wang X et al. (2019) inoculated soil with Ralstonia phages (10^6^ PFU/mL) and found that they significantly reduced the *R. solanacearum* population, with a control efficacy of 83.4% against tomato BW.

Due to the strong host specificity of phages, their application mostly follows the principle of “isolating phages from farm soil and returning them to the farm” (Díaz-Muñoz and Koskella, 2014; Ye et al., 2019). Studies have shown that the combination of multiple phages effectively inhibits resistance development in *R. solanacearum* and improves the control efficacy of BW (Wang et al., 2024). In current reports on phage cocktail applications, most Ralstonia phages used in these combinations belong to the class Caudoviricetes. Wei et al. (2017) screened phage P1 combinations capable of lysing the host within a short period based on lysis kinetics, resulting in a 20% reduction in BW incidence; Magar et al. (2022) utilized a combination of Ralstonia phages RpT1 and RpY2, which exhibit a broad host range, to significantly reduce BW incidence. Therefore, the biological characteristics of phages, such as host range and lysis kinetics, are critical criteria for formulating effective phage cocktails (Gill and Hyman, 2010; Villalpando-Aguilar et al., 2022; Tang et al., 2024).

To construct a phage cocktail with good control efficacy on tobacco BW in different areas of Xiangxi Tujia Zu and Miao Zu Autonomous Prefecture, Hunan Province, China, this study isolated *R. pseudosolanacearum* and its phages from tobacco fields with a high BW incidence in this region. After comparing the host range and single phage control efficacy, four phages were selected to construct a phage cocktail. Pot experiments showed that phage cocktails improved the control efficacy of BW. This study provides high-quality candidate resources for the biological control of BW.

## 2 Materials and methods

### 2.1 Isolation, purification, and identification of *R. pseudosolanacearum* and phages

*Ralstonia pseudosolanacearum* strains were isolated from tobacco plants with BW collected among towns in the Xiangxi Tujia Zu and Miao Zu Autonomous Prefecture (Xiangxi Prefecture), Hunan Province. *Ralstonia pseudosolanacearum* strains were obtained using the plate streaking method on nutrient broth (NB) medium (10 g tryptone, 3 g beef extract, 10 g glucose, and 5 g NaCl, 1000 mL ddH_2_O) and identified using 16S rRNA gene sequencing and strain-specific PCR (759/760) (Wicker et al., 2007). Lytic phages were isolated from tobacco rhizosphere soil using the isolated *R. pseudosolanacearum* strains as hosts and employing the modified double-layer agar method, in which 1 g of soil was added to 10 mL of sterile water, vortexed, and centrifuged at 12,000 rpm for 10 min. The supernatant was filtered through a 0.22-μm bacterial filter (Millex, Tullagreen, Carrigtwohill, Co. Cork., Ireland). Equal volumes of NB medium and 0.3% host bacterial suspension (V/V) were added to the filtrate and co-cultured at 30°C for 12 h. The culture was centrifuged and filtered, and the filtrate was diluted 1000-fold with SM buffer (50 mM Tris-CL, pH=7.5, 100 mM NaCl, 10 mM MgSO_4_, and 0.01% gelatin solution). The diluted solution was mixed with an equal volume of the *R. pseudosolanacearum* suspension (OD_600_ = 1.0), comprising the top layer of the plate, which was incubated at 37°C until plaques appeared. This process was repeated more than five times to complete phage purification (Kropinski et al., 2009).

### 2.2 Phage host range determination

Host range determination experiments were conducted using 38 *R. pseudosolanacearum* strains, among which 31 were isolated from tobacco (RStab-1 to RStab-31), 2 from peppers (RSpep-1, RSpep-2), 2 from potatoes (RSpot-1, RSpot-2), and 3 from peanuts (RSpea-1, RSpea-2, RSpea-3) (**Supplementary Table S1**). Each *R. pseudosolanacearum* strain was mixed with NB solid medium (0.2%, V/V) at 45°C and poured into plates. After the medium solidified, 5 μL of phage was added to the plate surface, spread evenly, and incubated overnight at 30°C. Plaque transparency was observed, and the host range was recorded. *Ralstonia pseudosolanacearum* strains with clear plaques were selected for subsequent pot inoculation experiments (Wang et al., 2022).

### 2.3 Pot experiments for the control of tobacco BW with phages

Yunyan 87 tobacco plants at the four-leaf stage were transplanted into a seedling substrate (Hunan Xianghui Agricultural Technology Development Co., Ltd., China). The pathogen used to inoculate tobacco was selected from the *R. pseudosolanacearum* strain, which was lysed using all nine phages in section 2.2. The pathogen was adjusted to OD_600_ = 0.1 with phosphate-buffered saline (PBS: 137 mM NaCl, 2.7 mM KCl, 10 mM Na_2_HPO_4_, and 1000 mL ddH_2_O), and the phage titer was adjusted to 10^8^ PFU/mL. Each plant was first inoculated with 10 mL of the *R. pseudosolanacearum* suspension (OD_600_ = 0.1), followed by 50 mL of the phage suspension. The experimental design comprised 12 individual tobacco plants per treatment with 3 experimental replicates, the temperature of the greenhouse was kept at 30°C throughout the experiment. The disease incidence was investigated and recorded for each plant at the early and peak stages of disease development. The disease index (DI) and control efficacy (CE) were calculated (Huang et al., 2024) as follows:


(1) DI=100×(1×n1+3×n3+5×n5+7×n7+9×n9)/(n×9)

(2) CE=(C−T)/C×100%

Where DI is the disease index; 1–9 refers to different disease classification levels; n1–n9 is the number of infected plants in each disease classification level; n is the number of plants investigated; CE is the control efficacy (T versus C); C is the disease index of the control group; and T is the disease index of the treatment group.

### 2.4 Construction and efficacy evaluation of the phage cocktail

Lytic Ralstonia phages were divided into two groups based on the host range and control efficacy. Two phages were selected from each group to construct five phage cocktails following the principle of ‘broader host range + higher control efficacy’: BPC-1 (YL3, YL4), BPC-2 (YL1, YL2, YL3, YL4), BPC-3 (YL1, YL3, YL4), BPC-4 (YL1, YL4), and BPC-5 (YL2, YL3). Adjust all phage titers to 1×10^8^ PFU/mL using PBS buffer, then combine the phages in equal proportions according to the cocktail combination to ensure a consistent final titer of 1×10^8^ PFU/mL in each cocktail. To simulate the infection of plants with different *R. pseudosolanacearum* strains under natural conditions (Genin and Denny, 2012), three virulent *R. pseudosolanacearum* strains (RStab-5, RStab-12, and RStab-19) were mixed for inoculation (**Supplementary Figure S1**). Pot experiments were conducted to evaluate the control efficacy of the five phage cocktails.

### 2.5 Biological characteristics and lysis curves

For the temperature tolerance experiments, 1 mL of phage culture with an initial phage titer of 1×10^9^ PFU/mL was subjected to water bath treatment at different temperatures (30, 37, 50, 60, 70, 80, and 90°C) for 1 h and then cooled to room temperature. The plaques number were determined in the double-layer plate method to compare the phage titers after different temperature treatments. For the pH tolerance experiments, 10 μL of phage culture with an initial phage titer of 1×10^9^ PFU/mL was added to 990 μL of SM buffer with different pH values (1.0, 2.0, 3.0, 4.0, 5.0, 6.0, 7.0, 8.0, 9.0, 10.0, 11.0, 12.0, and 13.0) and treated in a water bath at the optimal temperature for 1 h. The pH was monitored using pH test strips (JINLIDA, Tianjin Jinda Chemical Reagent Co., Ltd., China) after the experiment to verify the stability of acid–base conditions throughout the experimental process. Phage titers after different pH treatments were determined using the double-layer plate method. For optimal multiplicity of infection (MOI) determination, phages and host bacteria (0.3%, V/V) were used for inoculation and cultured overnight, and the host bacterial and phage concentrations were determined. They were then mixed at MOI = 10^3^, 10^2^, 10^1^, 1, 10^−1^, 10^−2^, and 10^−3^ (**Table 1**) and incubated at the optimal temperature and pH for 6 h. The phage titers were determined at different ratios using the double-layer plate method (Huang et al., 2022; Tian et al., 2022). Equal proportions of the phage culture and host were added to 48-well plates at the optimal MOI and co-cultured at the optimal temperature for 12 h. The OD_600_ values of the co-culture were measured after 0–12 h using a microplate reader (TECAN spark, Tecan (Shanghai)Trading Co., Ltd., Shanghai) to plot the lysis curves (Wei et al., 2017).

**Table 1:** Phage MOI rationing.

MOI (Phage/Host)	10^−3^	10^−2^	10^−1^	1	10^1^	10^2^	10^3^
Phage (PFU/mL)	10^5^	10^5^	10^5^	10^5^	10^5^	10^5^	10^5^
Host bacteria (CFU/mL)	10^8^	10^7^	10^6^	10^5^	10^4^	10^3^	10^2^

### 2.6 Electron microscopy observation of phage morphology

Each phage culture (1×10^8^ PFU/mL) was concentrated using a 100-kDa ultrafiltration tube (Millipore, Tullagreen, Carrigtwohill, Co. Cork., Ireland), and 20 μL of concentrated phage solution was dropped onto a copper grid and allowed to settle naturally for 15 min. The excess liquid was removed with filter paper, and 20 μL of 2% phosphotungstic acid was added and left for 5 min for staining. After drying, four phages were observed and photographed using a Hitachi transmission electron microscopy (HT7800, Hitachi America Ltd., Japan) (Ahmad et al., 2021).

### 2.7 Genome sequencing and assembly

Each phage suspension was concentrated using 100-kDa ultrafiltration tubes (Millipore, Tullagreen, Carrigtwohill, Co. Cork., Ireland). DNase I (1 μg/mL, TransGen Biotech, TransGen Biotech Co., Ltd., Beijing) and RNase A (1 μg/mL, TransGen Biotech) were used to digest possible host nucleic acids in the suspensions and inactivated using water bath treatment at 75°C for 30 min. Phage genomes were extracted using a Virus DNA/RNA Extraction Kit (Beijing Tiangen DP-315). DNase I, RNase A, and EcoRI (New England Biolabs, Inc) were used to determine the nucleic acid type of the phages (Wilcox et al., 1996).

Whole genome sequencing was performed on the Illumina NovaSeq platform. The original sequencing data were quality controlled using FastQC and quality trimmed using Trimmomatic (Bolger et al., 2014). The A5-MiSeq and SPAdes *de novo* assembly methods were used to obtain complete phage genome sequences (Bankevich et al., 2012; Coil et al., 2015).

### 2.8 Comparative genomic analysis

GeneMarkS and RAST were used to predict the open reading frames (ORFs) in phage genomes (Besemer and Borodovsky, 2005; Aziz et al., 2008). For functional annotation, Diamond was used to compare the predicted protein sequences with the NCBI Non-Redundant (NR) database (Buchfink et al., 2015). Gene Ontology (GO) term annotation was performed using Blast2GO (Conesa et al., 2005).

Skani was used to calculate the average nucleotide identity (ANI) between phage genomes, and heat maps were generated to visualize genome similarities (Shaw and Yu, 2023). Phylogenetic analysis was performed using the Mashtree method based on Mash distances (Katz et al., 2019). Mash was used to calculate Mash distances between phage genomes (Ondov et al., 2016), and Mashtree was used to construct a phylogenetic tree based on Mash distances, with the kmer set to 21 and sketch set to 1000. iTOL was used to visualize and annotate the phylogenetic tree (Letunic and Bork, 2021). To identify genome structure conservation and variation, Easyfig 2.2.5 was used for collinearity analysis of phage genome sequences (Sullivan et al., 2011).

### 2.9 Tail fiber protein structure and function analysis

Jalview was used to visualize the alignment results and identify conserved and variable regions (Sievers and Higgins, 2018; Procter et al., 2021). AlphaFold 3 was used for three-dimensional structure prediction of tail fiber protein amino acid sequences (Abramson et al., 2024). To verify the AlphaFold prediction results, SWISS-Model was used for homology modeling (Waterhouse et al., 2018). PyMOL was used for visualization analysis and coloring of the predicted structures, focusing on analysis the tip domain, which may affect host recognition (Rosignoli and Paiardini, 2022).

### 2.10 Statistical analysis

Analysis of variance (ANOVA) in SPSS was used to identify significant differences in the control efficacies of single phages and phage cocktails (p<0.05).

## 3 Results

### 3.1 Isolation and identification of *R. pseudosolanacearum* and phages, and construction of phage cocktails

#### 3.1.1 Isolation and identification of *R. pseudosolanacearum* and phages, evaluation of single-phage biocontrol potential, and construction of phage cocktails

This study collected tobacco with BW to screen phages with effective lytic activity against tobacco BW pathogens in Xiangxi Tujia Zu and Miao Zu Autonomous Prefecture, Hunan Province. A total of 26 *R. pseudosolanacearum* strains (RStab1–RStab26) and 9 phages (YL1–YL9) were isolated and purified (**Supplementary Figure S2**).

Host range analysis (**Supplementary Table S1**) showed that there were significant differences in the host ranges of nine phages against 38 *R. pseudosolanacearum* strains isolated from tobacco, peanut, pepper, and potato. YL1 and YL4 lysed 84.21 and 81.58% of the tested *R. pseudosolanacearum* strains, respectively, showing higher lysis rates than the other seven phages. Therefore, YL1 and YL4 were defined as broader host range phages. As all nine phages could lyse RStab-12 with obvious plaques, RStab-12 was selected to evaluate the biocontrol potential of individual phages.

**Figure 1:**
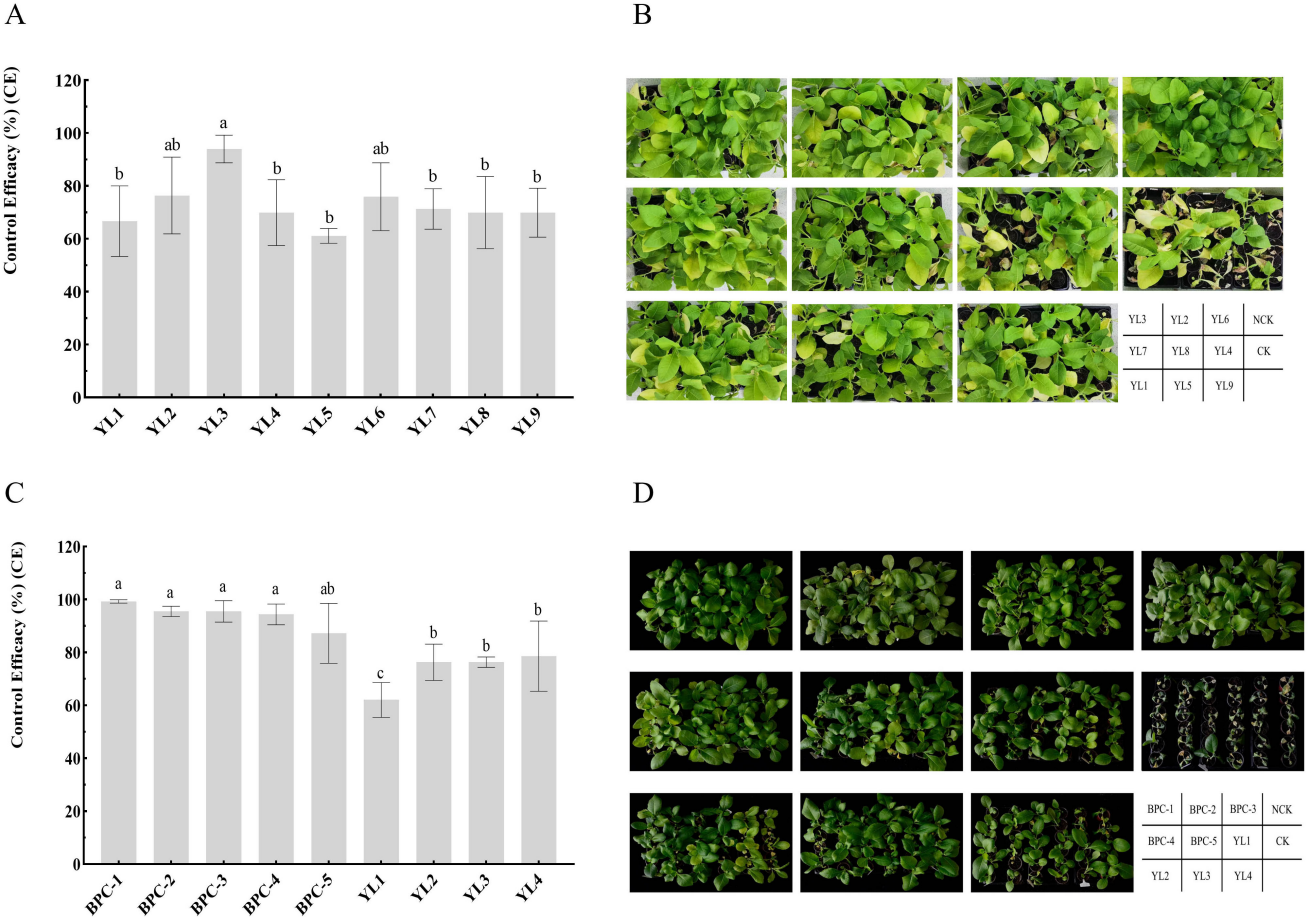
Control efficiency of phages and phage cocktails against BW in pots. **(A, B)** show the evaluation of single-phage biocontrol potential; **(C, D)** show the control efficacy of phage cocktails against tobacco BW inoculation with three *R. pseudosolanacearium* strains. Letters in the bar chart indicate significant differences according to Duncan’s analysis (P ≤ 0.05); NCK is the negative control group.

Pot experiments were conducted to compare the control efficacies of nine individually inoculated phages against tobacco BW to evaluate their biocontrol potential. The survey showed that at 7 days after inoculation with RStab-12, seedlings in the control treatment (CK) entered the peak period of BW, while only partial wilting was observed in the phage-treated groups at the same time. At 14 days after inoculation, the DI of CK were significantly higher than that of the phage-treated groups. Among them, YL3 had the highest control effect on BW (93.98 ± 3.03%), followed by YL2 (76.39 ± 8.37%), and YL9 had the lowest control efficacy (61.11 ± 1.60%) (**Figures 1A, B**). This indicates that phage inoculation effectively reduces the occurrence of BW but that there are large differences in efficacy between phages.

The two phages with the highest control efficacy (YL2 and YL3) were selected and combined with YL1 and YL4 to construct five phage cocktails: BPC-1 (YL3, YL4), BPC-2 (YL1, YL2, YL3, YL4), BPC-3 (YL1, YL3, YL4), BPC-4 (YL1, YL4), and BPC-5 (YL2, YL3). Further research on YL1, YL2, YL3, and YL4 was conducted to clarify their biological characteristics and taxonomic relationships.

#### 3.1.2 Control efficacy of phage cocktails against tobacco BW inoculation with three *R. pseudosolanacearium* strains

Using pot experiments, the control efficacy of five cocktails and four single phages was compared against mixed inoculation with three *R. pseudosolanacearum* strains of high virulence (**Supplementary Figure S1**). At 14 days after inoculation, the survey results showed that the control efficacies of all five phage cocktails against BW were above 87%. BPC-1 exhibited the highest control efficacy (99.25 ± 0.65%), and the control efficacies of BPC-2, BPC-3, BPC-4, and BPC-5 were 95.49 ± 1.95, 95.49 ± 4.06, 94.36 ± 3.90, and 87.22 ± 11.30%, respectively. The control efficacy of each phage was between 62.03 ± 6.60 and 78.57 ± 13.30%. Comparisons between phage cocktails and individual phages showed no significant differences among the five cocktails. However, the control efficacies of BPC-1, BPC-2, BPC-3, and BPC-4 were significantly higher than those of the individual phages; all phage cocktails achieved control efficacies above 94% against BW. The combination of two high control efficacy phages (YL2 and YL3) in a cocktail (BPC-5) exhibited a control efficacy of 87.22 ± 11.30%, which was significantly improved compared to that of phage YL1. BPC-5 had a 10.9% increase in control efficacy compared to YL2 and YL3. The addition of two broader host range phages (YL1 and YL4) to BPC-5 (forming BPC-2) improved the control efficacy against BW by 8.27% (**Figures 1C, D**). The experimental results indicate that combining phages with a broad host range and high control efficacy enhances their coverage against *R. pseudosolanacearum* strains and improves their control efficacy against BW.

### 3.2 Biological characteristics

**Figure 2:**
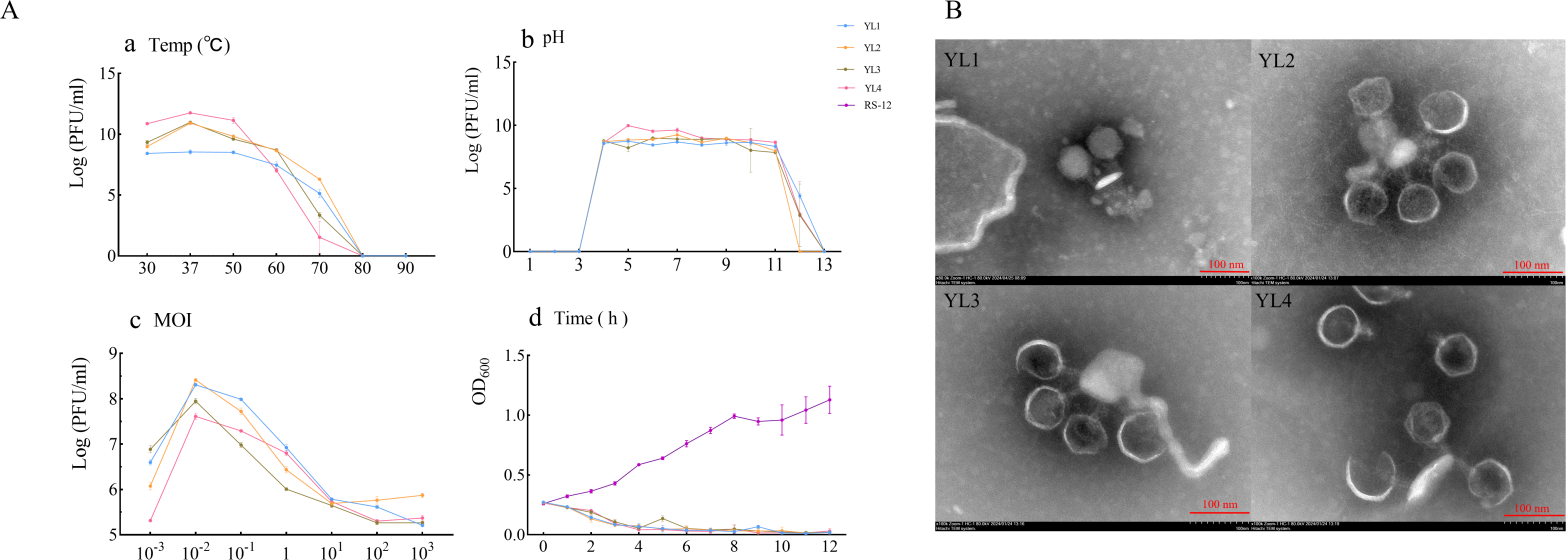
Biological characteristics, lysis curves, and transmission electron microscopy showing the morphology of four phages. **(A-a–A-d)** show temperature sensitivity, pH stability, MOI, and lysis curves, respectively of four phages; **(B)** shows the morphology of four phages observed using transmission electron microscopy.

Phage stability is affected by environmental factors, such as temperature and pH. Temperature sensitivity experiments showed that YL1’s titer remained stable in the range of 30–50°C but decreased significantly at temperature greater than 60°C. The titers of YL2, YL3, and YL4 remained stable in the range of 30–60°C but decreased significantly after water bath treatment at 70°C for 1 h, with YL2 showing a smaller decrease than YL3 and YL4. No plaques were detected for the four phages at 80 or 90°C (**Figure 2A-a**), indicating that 80°C was the lethal temperature.

pH stability experiments showed that the titers of all four phages remained stable above 10^7^ PFU/mL in the pH range of 4–11 but decreased significantly at pH 11. No plaques were detected at pH 3. At pH 12, YL2 showed no plaque, while the titers of YL1, YL2, and YL3 decreased to 10^4^ PFU/mL. No plaques were detected at pH 13 (**Figure 2A-b**). These results indicate that the four phages have substantial application potential under environmental conditions of 30–50°C and pH 4–11.

The optimal MOI for all four phages was 10^−2^ (**Figure 2A-c**). The lysis curves of phages against RStab-12 were determined at the optimal MOI. After combining the four phages, the OD_600_ of the co-culture decreased to less than 0.074 within 4 h. Notably, the OD_600_ of YL3 increased to 0.135 at 5 h and decreased again to 0.053 after 1 h. In CK (RStab-12), the OD_600_ of CK continued to increase within 12 h, reaching 1.128 at 12 h (**Figure 2A-d**). The experimental results showed that an MOI = 10^−2^ enabled all four bacteriophages to produce more progeny and lyse the host within 4 h.

Transmission electron microscopy observation revealed that all four phages had large icosahedral heads (YL1: 72.03 ± 6.50 nm; YL2: 70 ± 0.82 nm; YL3: 71 ± 0.82 nm; YL4: 72.67 ± 1.25 nm) and relatively short tails (YL1: 31.33 ± 1.70 nm; YL2: 32 ± 2.83 nm; YL3: 30 ± 2.45 nm; YL4: 29.67 ± 1.25 nm). (**Figure 2B**).

### 3.3 Genomic analysis

#### 3.3.1 Genome characteristics and phylogenetic analysis

The nucleic acids of YL1, YL2, YL3, and YL4 were not digested by RNase A but were all cleaved into DNA fragments of different sizes by EcoRI (**Supplementary Figure S3**), indicating that they were all double-stranded DNA phages. Genome sequencing also confirmed that they were all double-stranded circular DNA phages, with genome lengths of 59,600, 60,770, 61,339, and 60,673 bp, respectively, G + C contents of 64.52, 64.86, 64.92, and 65.02%, respectively, and 73, 73, 75, and 74 ORFs, respectively (**Supplementary Tables S2**-**S5**). The genome sequences of YL1, YL2, YL3, and YL4 were submitted to GenBank under accession numbers PQ295876, PQ295877, PQ295878, and PQ295879, respectively. BLASTn analysis showed that YL1, YL2, YL3, and YL4 had more than 92% similarity with the genome sequences of previously reported *Gervaisevirus* phages in the Caudoviricetes class, such as QKW1 (GenBank accession no. PP236328), AhaGv (GenBank accession no. OR820515), P2110 (GenBank accession no. OP947226), and GP4 (GenBank accession no. MH638294), indicating that they belong to this genus and class.

**Figure 3:**
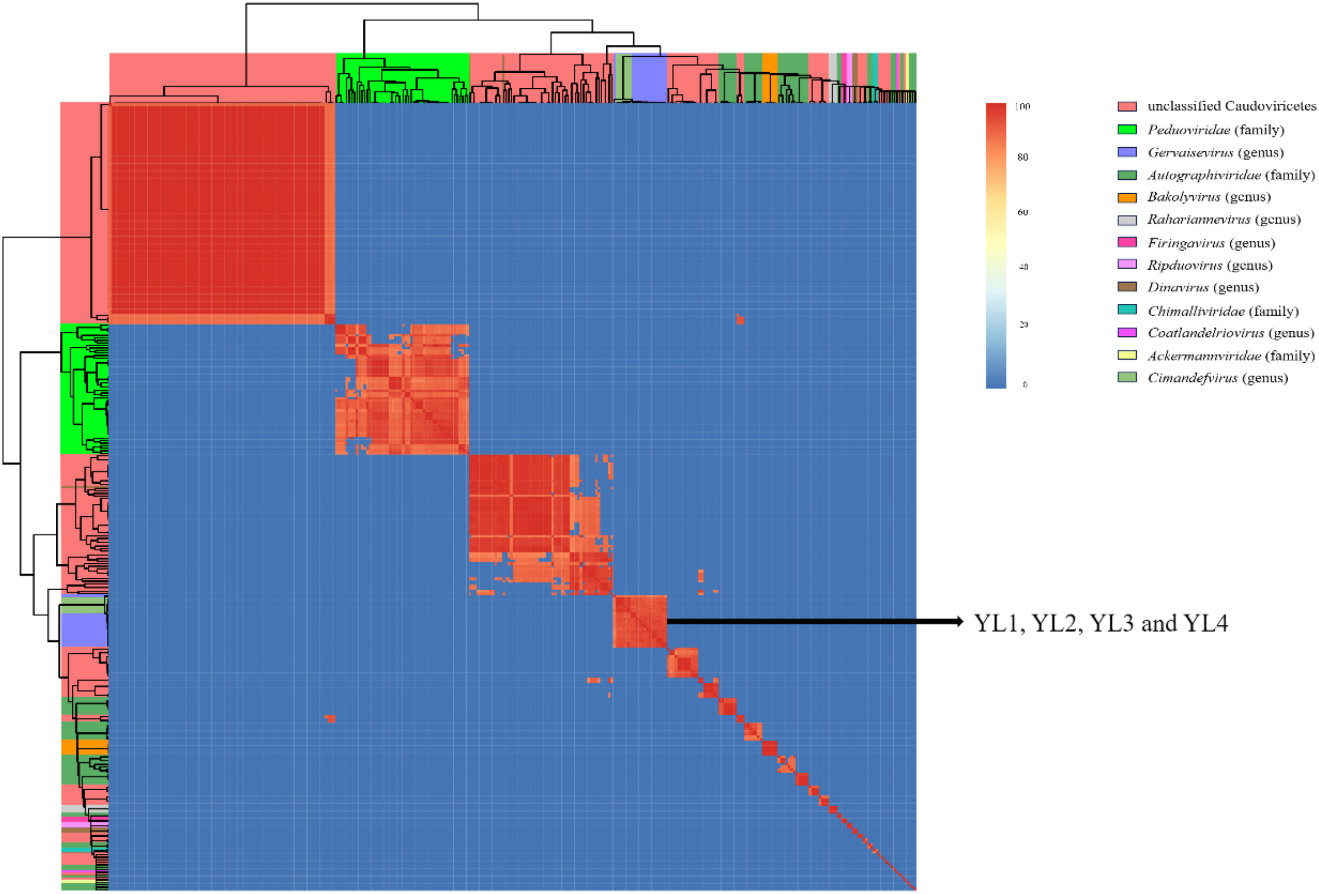
Nucleotide similarity heatmap of four phages with 317 Ralstonia phages in the Caudoviricetes class. The ANI between phage genomes was calculated using Skani and visualized as a heatmap. Different colored squares on the top and left sides of the heatmap represent different taxonomic relationships among the phages. The color intensity in the heatmap indicates the degree of nucleotide similarity between phage genomes.

The genome sequences of these four phages were compared with 317 Ralstonia phages of the Caudoviricetes class recorded in the NCBI database. These 321 phages were divided into four families and eight genera according to their evolutionary relationships. YL1, YL2, YL3, and YL4 were all located in the *Gervaisevirus* branch (**Supplementary Figure S4**), showing high similarity with other members of this genus. All four phages were identified as belonging to the *Gervaisevirus* genus of the Caudoviricetes class based on the classification principles of the Bacterial and Archaeal Viruses Subcommittee (BAVS) (Adriaenssens and Brister, 2017). Skani was used to calculate the ANI between phage genomes to further quantify the differences between genomes. The ANI values of YL2, YL3, and YL4 were all above 95%, indicating that they belong to the same species, while the ANI values between YL1 and YL2, YL3, and YL4 ranged from 92 to 94%, indicating that they are not the same species (**Figure 3**).

#### 3.3.2 Protein function annotation and comparative genome analysis

Annotation results of the four phage genomes showed that the three functional proteins in YL1 and YL3 did not have similar proteins in the NR protein database (BLASTp e-value less than 1e−5), while YL2 and YL4 each had four. YL1, YL2, YL3, and YL4 had 36, 38, 41, and 54 hypothetical proteins, respectively. ORF2 occupied a large proportion of their genomes (YL1: 5.32%, YL2: 7.29%, YL3: 7.29%, YL4: 7.29%), and annotation showed that ORF2 was homologous to *DarB* (defense against restriction), which is required to protect foreign genomic DNA from restriction by host type I R-M systems (Piya et al., 2017).

This study classified the ORFs of the four phages into four functional types: lysis, morphogenesis, replication and regulation, and packaging genes. Among the ORFs related to lysis, all four phages had holin proteins. ORFs related to morphogenesis mainly encoded head proteins, tail fiber proteins, virion structural proteins, and portal proteins. ORFs related to replication and regulation encoded functional proteins, such as RecE-like recombination exonuclease and plasmid-derived single-stranded DNA-binding protein. ORFs related to packaging encoded functional proteins such as terminase small subunit and phage terminase large subunit (**Supplementary Tables S2**-**S5**).

**Figure 4:**
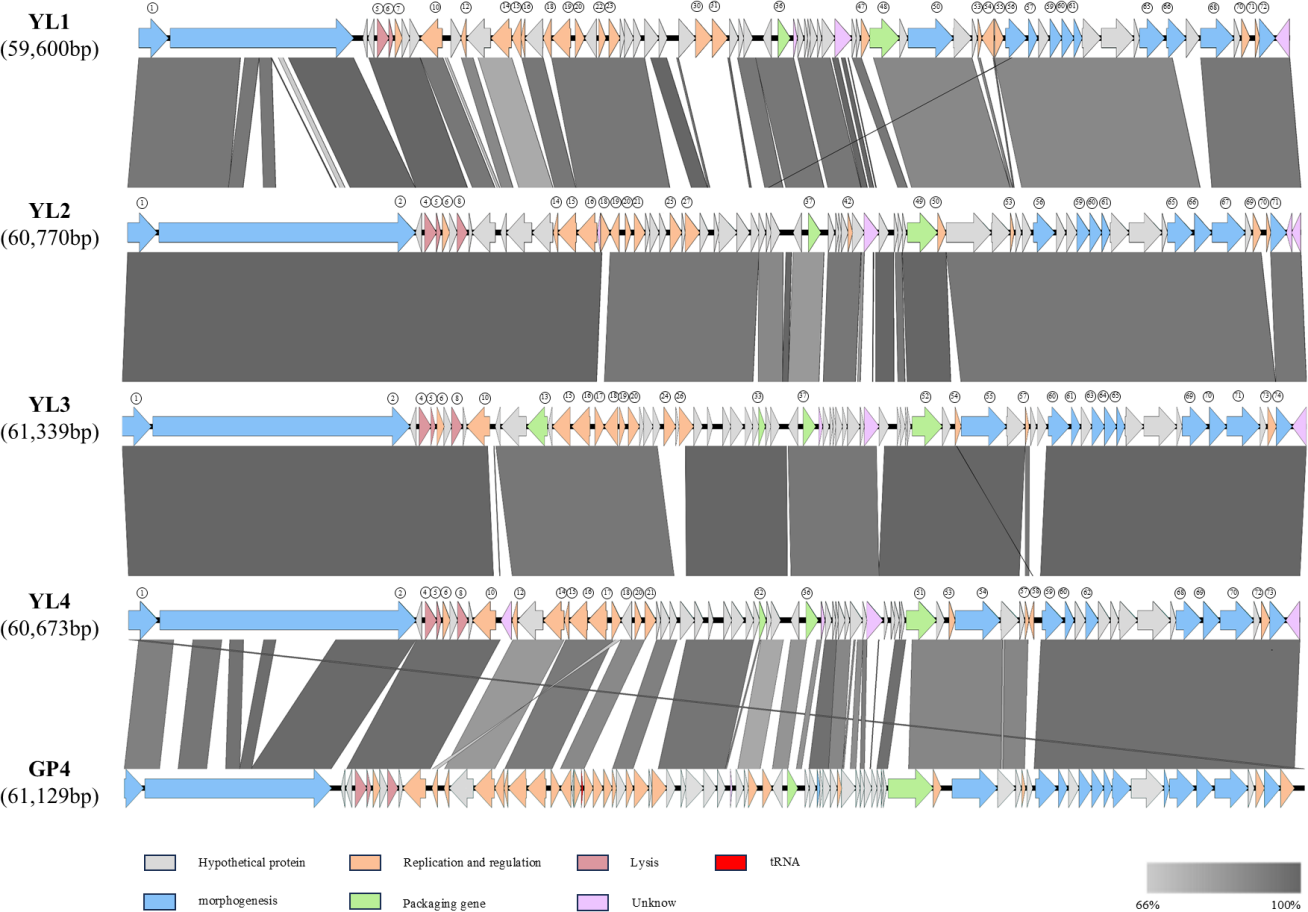
Comparative genome of four phages. Comparison of whole genome similarity between YL1, YL2, YL3, and YL4. Arrows represent transcription direction of genes, and genes with consistent functional annotations are represented by the same color. Gray bands between genomes indicate amino acid similarity.

Easyfig was used for comparative genome analysis of the four phage genomes, which showed good consistency with GP4 (Wang R et al., 2019). The arrangement positions and transcription directions of most genes with the same functions in the genome were consistent, but there were partial gene deletions and position shifts between phages. Compared to the other three phages, YL1 had nucleic acid sequence deletions in ORF2, significant differences in ORF11, ORF30-31, and ORF67 compared to YL2, and position shifts in ORF55. ORF56 in YL1 showed gene shifts in YL2, and YL2, YL3, and YL4 showed high genome consistency, with ORF54 in YL3 and ORF58 in YL4 having position shifts (**Figure 4**).

#### 3.3.3 Structure and function prediction of tail fiber proteins

**Figure 5:**
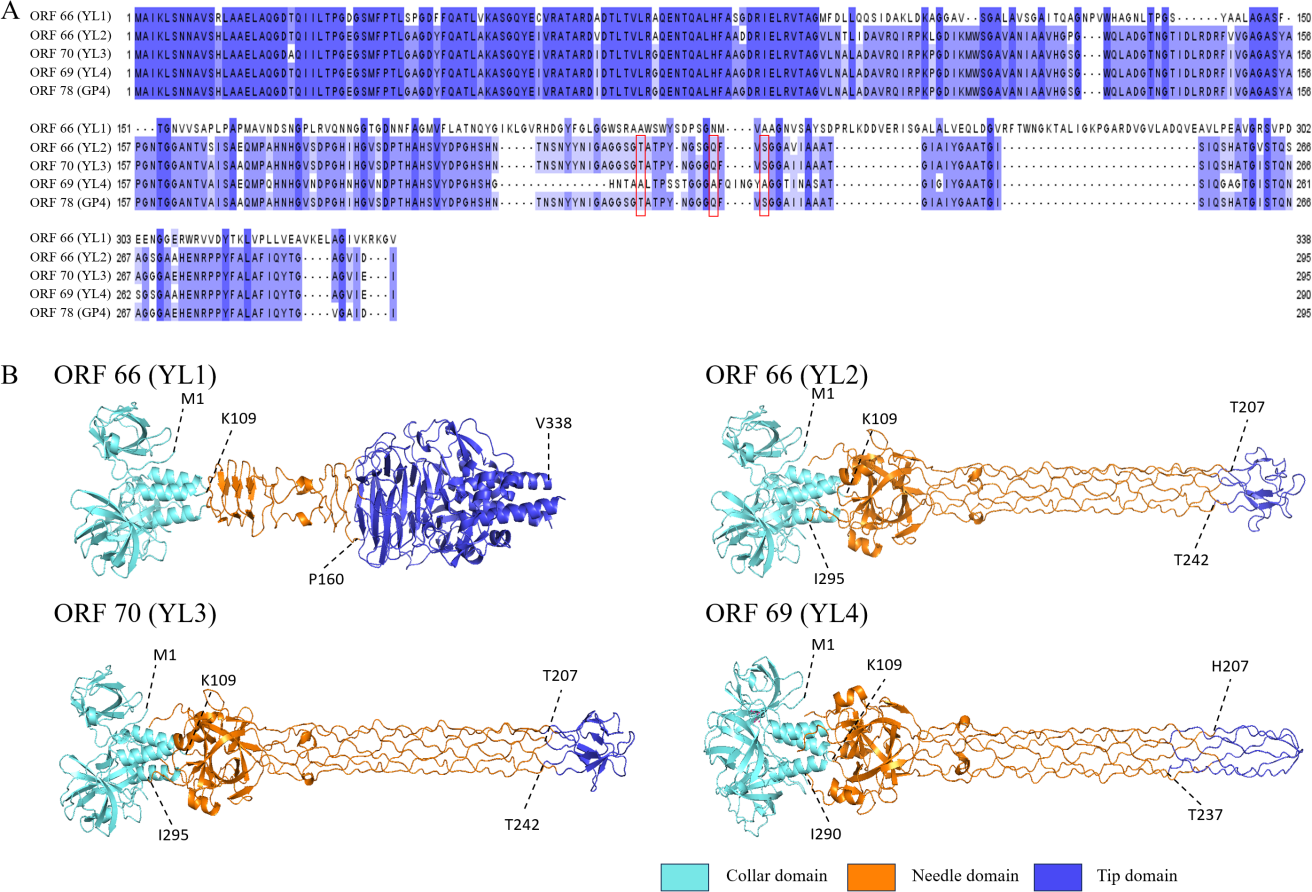
Amino acid sequence similarity and three-dimensional structure prediction of tail fiber protein II. **(A)** shows amino acid sequence similarity of tail fiber protein II. Red boxes indicate mutations of polar amino acids in ORF66 (YL2), ORF70 (YL3) and ORF78 (GP4), and deeper blue indicates higher conservation. **(B)** shows predicted structures of tail fiber II proteins of four phages using Alphafold 3. Light blue areas are collar domains located at the base of phage tail fibers; orange areas are needle domains located in the middle long part of tail fibers; dark blue areas are tip domains located at the “tip” part of the end structure of phage tail fiber II; and dotted lines with numbers indicate connections between structural domains.

Host range analysis showed that the four phages had significant differences in lysis capacity against 38 *R. pseudosolanacearum* strains. Since phage tail fiber proteins play a key role in host recognition and infection processes, analysis found that all four phages had two types of tail fiber proteins: tail fiber protein I (ORF65 (439 aa, YL1), ORF65 (439 aa, YL2), ORF69 (439 aa, YL3), ORF68 (439 aa, YL4)), and tail fiber protein II (ORF66 (338 aa, YL1), ORF66 (295 aa, YL2), ORF70 (290 aa, YL3), ORF69 (290 aa, YL4)). Based on amino acid sequence alignment using Jalview, the amino acid sequences of tail fiber protein I had high consistency in the four phages, while the C-terminal amino acid sequences of tail fiber protein II showed significant differences (**Figure 5A**). Therefore, highly conserved tail fiber protein I is expected to have little effect on host recognition, while tail fiber protein II may play an important role in host recognition.

AlphaFold 3 was used to predict the three-dimensional structure of tail fiber protein II from four phages. The visualization of the predicted structure revealed that tail fiber protein II adopts a trimeric structure. Furthermore, the N-terminal structure of tail fiber protein II was highly consistent with that of ORF78 (GP4) (Zheng et al., 2023). However, the “tip domain” (P160-V338) of ORF66 (YL1) was significantly different from the “tip domains” of ORF66 (T207-T242, YL2), ORF70 (T207-T242, YL3), and ORF69 (H207-T237, YL4), with 11 β-sheets and 5 α-helices. However, the “tip domains” of ORF66 (YL2), ORF70 (YL3) and ORF69 (YL4) had similar structures (**Figure 5B**). In addition, the ORF66 (YL1) “tip domain” region also had more aromatic amino acids, such as phenylalanine (F), tryptophan (W), and tyrosine (Y), and positively charged amino acids, such as arginine (R) and lysine (K), which provide more binding sites for its interaction with hosts (Bartual et al., 2010; Vyas, 1991). The “tip domain” amino acid sequences of ORF66 (YL2), ORF70 (YL3), and ORF78 (GP4) had high consistency, but those of ORF69 (YL4) had a large number of amino acid deletions and mutations, including seven deleted polar amino acids (T207, N208, S209, N210, Y211, Y212, N213) and three polar amino acids mutated to non-polar amino acids (T221A, Q230A, S233A).

To further elucidate the differences in the “tip domains” between ORF66 (YL2), ORF70 (YL3), and ORF69 (YL4), protein surface electrostatic analysis was performed using Adaptive Poisson–Boltzmann Solver in PyMOL (APBS) (Jurrus et al., 2018). Five amino acid residues (G217, G218, S219, G220, and T221) on the “tip domain” surfaces of ORF66 (YL2) and ORF70 (YL3) carried negative charges, while that in ORF69 (YL4) carried more positive charges (G218, G219, G220, A221, and F222) (**Supplementary Figure S5**). More positive charges in the “tip domain” are believed to enhance the binding ability of YL4 to its host receptor, thereby potentially conferring a broader host range.

## 4 Discussion

Phage therapy is considered a most promising technology for controlling plant bacterial diseases (Kering et al., 2019; Pandit et al., 2022). *Ralstonia pseudosolanacearum* is a typical “species complex” with diverse genotypic variations. Due to the strong host specificity and narrow host range of most phages, mixing phages with different host ranges in phage cocktails can improve the efficacy of phage therapy or biocontrol (Buttimer et al., 2017; Magar et al., 2022). This study isolated 26 *R. pseudosolanacearum* strains and 9 lytic phages from Xiangxi Tujia Zu and Miao Zu Autonomous Prefecture, Hunan Province. By comparing the host range and pot control efficacy of phages, four were selected to construct five phage cocktails. The control efficacy of combined phages against mixed inoculation with *R. pseudosolanacearum* was significantly higher than that of individual phages.

In field applications, complex soil environments affect phage lytic activity. Most reported phages are stable in the range of 28–50°C and pH 5–10 (Magar et al., 2022; Wang et al., 2022; Lin et al., 2023; Huang et al., 2024). In this study, YL1, YL2, YL3, and YL4 maintained stable titers at 30–50°C and pH 4–11, enabling better adaptation to problems caused by changes in environmental temperature and soil pH, which may reduce phage activity or infection ability (**Figures 2A-a, A-b**).YL1, YL2, YL3, and YL4 had high lysis efficiency and rapidly reduced the *R. pseudosolanacearum* population within 4 h, thereby reducing disease occurrence (**Figure 2A-d**). Wei et al. (2017) summarized four types of phage lysis curves, including a mode that showed immediate growth inhibition of host bacteria, similar to the lysis curves of the four phages in this study. To delay host bacterial resistance to phages, phages with broad lysis spectra, high control efficacy, and short infection cycles should be used when constructing phage cocktails (Jones et al., 2007; Kaur et al., 2021). This study, building on the method established by Wei and Magar, concentrated on screening phages with a broad host range and high control efficacy, which were subsequently combined into a phage cocktail. The results demonstrated that, compared to individual phages, the phage cocktail significantly enhanced the control efficacy against a mixed inoculation of three *R. pseudosolanacearum* strain. BPC-1 exhibited the highest control effect (99.25% ± 0.65%). Subsequent field experiments should further compare the control efficacy of phage cocktails against BW in agricultural and ecological environments. In addition, through whole genome sequencing and AlphaFold 3 prediction, this study discovered the tail fiber II and its three-dimensional structure, hypothesizing that this structure provides the capability to bind with the outer membrane protein receptors of *R. pseudosolanacearum*. These findings provide valuable materials for further research into the interaction mechanisms between phages and their host receptors.

Whole genome sequencing provides critical insights into the taxonomic relationships and genomic characteristics of bacteriophages (Dion et al., 2020). According to genomic analysis and BAVS classification principles (Adriaenssens and Brister, 2017), the four Ralstonia phage strains belonged to the *Gervaisevirus* genus within the Caudovirivetes class, demonstrating high similarity (>92%) with other members of this genus. Functional annotation revealed that the genomes of the four Ralstonia phage strains contained four functional types, namely lysis, morphogenesis, replication and regulation, and packaging genes, consistent with previously reported Gervaisevirus phages GP4 and P2110 (Wang et al., 2019; Chen et al., 2023). Comparative genome analysis revealed variations in gene arrangement, even among genomically similar phages. These variations suggest dynamic genomic rearrangement and gene mutation processes potentially driven by horizontal gene transfer and evolutionary selective pressures, which are critical mechanisms underlying bacteriophage genomic diversity (Brüssow and Hendrix, 2002). Overall, the genetic diversity and functional predictions for these four bacteriophage strains highlight the conserved characteristics and plasticity of bacteriophages in the *Gervaisevirus* genus.

The abundant lipopolysaccharides (LPS) and outer membrane proteins in bacteria are the main binding sites for phages. The interactions between phage tail fiber proteins and bacterial receptors determine the phage’s host range. The number of positively charged amino acids and aromatic amino acids in the “tip domain” of tail fiber proteins affects the host range and adsorption ability of phages (Nobrega et al., 2018; Mourosi et al., 2022). The “tip domain” of tail fiber protein II of ORF66 (YL1) had more aromatic amino acids and positively charged amino acids, which interact with rough LPS and negatively charged phospholipids in the host outer membrane, respectively (Vyas, 1991; Raetz and Whitfield, 2002; Bartual et al., 2010; Rakhuba et al., 2010). Phages that bind rough LPS usually have a broader host lysis range, which potentially explains the broader host range of YL1. The amino acid sequences of the “tip domains” of ORF66 (YL2) and ORF70 (YL3) were highly similar, with amino acid mutations (S228G, V237I) in ORF70 (YL3), which reduced its lysis rate by 13.16%. Compared to ORF69 (YL4), the amino acid deletions and mutations in ORF66 (YL2) and ORF70 (YL3) may reduce the binding ability of tail fiber II to the host receptor site (Bolen and Rose, 2008). Further, the “tip domain” of tail fiber protein II of ORF69 (YL4) also contained a positively charged histidine (H207), which enhanced its electrostatic adsorption ability to the host outer membrane (Bartual et al., 2010), increasing the host range of YL4 (**Figure 5B**). Subsequent studies should verify these speculations by performing amino acid mutations on the “tip domain” of tail fiber protein II.

## 5 Conclusion

Phage therapy shows promising biocontrol potential for managing BW caused by *R. pseudosolanacearum* in agricultural production. This study isolated nine phages and selected those with a broader host range (YL1 and YL4) and high control efficacy (YL2 and YL3) to construct five cocktails. In pot experiments, BPC-1 (YL3 and YL4) exhibited the highest control efficacy (99.25%). The four phages maintained stable titers at 30–50°C and pH 4–11, demonstrating substantial thermal stability and pH tolerance. Whole genome sequencing revealed that phages YL1, YL2, YL3, and YL4 belonged to the genus *Gervaisevirus*. AlphaFold 3 prediction of the three-dimensional structures of tail fiber protein II in the four phages showed that ORF66 (YL1) had a distinct structure in the “tip domain” compared to the other three phages, with more aromatic amino acids and positively charged amino acids. ORF70 (YL3), ORF66 (YL2), and ORF69 (YL4) had similar structures, but ORF69 (YL4) had more amino acid mutations and deletions and more positive charges in the tip region, potentially explaining their different host ranges.

## Data Availability

The datasets presented in this study can be found in online repositories. The names of the repository/repositories and accession number(s) can be found in the article/**Supplementary Material**.

## References

[B1] Abramson J. Adler J. Dunger J. Evans R. Green T. Pritzel A.. (2024). Accurate structure prediction of biomolecular interactions with AlphaFold 3. Nature 630, 493–500. doi: 10.1038/s41586-024-07487-w 38718835 PMC11168924

[B2] Adriaenssens E. Brister J. R. (2017). How to name and classify your phage: an informal guide. Viruses 9 (4), 70. doi: 10.3390/v9040070 28368359 PMC5408676

[B3] Ahmad A. A. Addy H. S. Huang Q. (2021). Biological and molecular characterization of a jumbo bacteriophage infecting plant pathogenic *Ralstonia solanacearum* species complex strains. Front. Microbiol. 12. doi: 10.3389/fmicb.2021.741600 PMC8504454 34646257

[B4] Askora A. Kawasaki T. Fujie M. Yamada T. (2017). Lysogenic conversion of the phytopathogen *Ralstonia solanacearum* by the P2virus ϕRSY1. Front. Microbiol. 8. doi: 10.3389/fmicb.2017.02212 PMC5694545 29184542

[B5] Aziz R. K. Bartels D. Best A. A. DeJongh M. Disz T. Edwards R. A.. (2008). The RAST Server: rapid annotations using subsystems technology. BMC Genomics 9, 75. doi: 10.1186/1471-2164-9-75 18261238 PMC2265698

[B6] Bankevich A. Nurk S. Antipov D. Gurevich A. A. Dvorkin M. Kulikov A. S.. (2012). SPAdes: a new genome assembly algorithm and its applications to single-cell sequencing. J. Comput. Biol. 19, 455–477. doi: 10.1089/cmb.2012.0021 22506599 PMC3342519

[B7] Bartual S. G. Otero J. M. Garcia-Doval C. Llamas-Saiz A. L. Kahn R. Fox G. C.. (2010). Structure of the bacteriophage T4 long tail fiber receptor-binding tip. Proc. Natl. Acad. Sci. U.S.A. 107, 20287–20292. doi: 10.1073/pnas.1011218107 21041684 PMC2996694

[B8] Besemer J. Borodovsky M. (2005). GeneMark: web software for gene finding in prokaryotes, eukaryotes and viruses. Nucleic Acids Res. 33, W451–W454. doi: 10.1093/nar/gki487 15980510 PMC1160247

[B9] Bolen D. W. Rose G. D. (2008). Structure and energetics of the hydrogen-bonded backbone in protein folding. Annu. Rev. Biochem. 77, 339–362. doi: 10.1146/annurev.biochem.77.061306.131357 18518824

[B10] Bolger A. M. Lohse M. Usadel B. (2014). Trimmomatic: a flexible trimmer for Illumina sequence data. Bioinformatics 30, 2114–2120. doi: 10.1093/bioinformatics/btu170 24695404 PMC4103590

[B11] Brüssow H. Hendrix R. W. (2002). Phage genomics: small is beautiful. Cell 108, 13–16. doi: 10.1016/s0092-8674(01)00637-7 11792317

[B12] Buchfink B. Xie C. Huson D. H. (2015). Fast and sensitive protein alignment using DIAMOND. Nat. Methods 12, 59–60. doi: 10.1038/nmeth.3176 25402007

[B13] Buttimer C. McAuliffe O. Ross R. P. Hill C. O'Mahony J. Coffey A. (2017). Bacteriophages and bacterial plant diseases. Front. Microbiol. 8. doi: 10.3389/fmicb.2017.00034 PMC5247434 28163700

[B14] Chen K. Guan Y. Hu R. Cui X. Liu Q. (2023). Characterization of the lysP2110-HolP2110 lysis system in Ralstonia solanacearum phage P2110. Int. J. Mol. Sci. 24 (12), 10375. doi: 10.3390/ijms241210375 37373522 PMC10299722

[B15] Coil D. Jospin G. Darling A. E. (2015). A5-miseq: an updated pipeline to assemble microbial genomes from Illumina MiSeq data. Bioinformatics 31, 587–589. doi: 10.1093/bioinformatics/btu661 25338718

[B16] Conesa A. Götz S. García-Gómez J. M. Terol J. Talón M. Robles M. (2005). Blast2GO: a universal tool for annotation, visualization and analysis in functional genomics research. Bioinformatics 21, 3674–3676. doi: 10.1093/bioinformatics/bti610 16081474

[B17] Denny T. P. (2000). *Ralstonia solanacearum*–a plant pathogen in touch with its host. Trends Microbiol. 8, 486–489. doi: 10.1016/s0966-842x(00)01860-6 11121751

[B18] Díaz-Muñoz S. L. Koskella B. (2014). Bacteria-phage interactions in natural environments. Adv. Appl. Microbiol. 89, 135–183. doi: 10.1016/B978-0-12-800259-9.00004-4 25131402

[B19] Dion M. B. Oechslin F. Moineau S. (2020). Phage diversity, genomics and phylogeny. Nat. Rev. Microbiol. 18, 125–138. doi: 10.1038/s41579-019-0311-5 32015529

[B20] Elphinstone J. G. Allen C. Prior P. Hayward A. C. (2005). The current bacterial wilt situation: a global overview. Bacterial Wilt the Disease & the Ralstonia Solanacearum Species Complex. Available online at: https://www.semanticscholar.org/paper/The-current-bacterial-wilt-situation%3A-a-global-Elphinstone-Allen/87ad9037d22c7dc6c6dc288405ac82d6793f9959.

[B21] Genin S. Denny T. P. (2012). Pathogenomics of the Ralstonia solanacearum species complex. Annu. Rev. Phytopathol. 50, 67–89. doi: 10.1146/annurev-phyto-081211-173000 22559068

[B22] Gill J. J. Hyman P. (2010). Phage choice, isolation, and preparation for phage therapy. Curr. Pharm. Biotechnol. 11, 2–14. doi: 10.2174/138920110790725311 20214604

[B23] Huang B. Ge L. Xiang D. Tan G. Liu L. Yang L.. (2024). Isolation, characterization, and genomic analysis of a lytic bacteriophage, PQ43W, with the potential of controlling bacterial wilt. Front. Microbiol. 15. doi: 10.3389/fmicb.2024.1396213 PMC11324598 39149212

[B24] Huang S. Tian Y. Wang Y. García P. Liu B. Lu R.. (2022). The broad host range phage vB_CpeS_BG3P Is able to inhibit clostridium perfringens growth. Viruses 14 (4), 676. doi: 10.3390/v14040676 35458406 PMC9033094

[B25] Ji M. Liu Z. Sun K. Li Z. Fan X. Li Q. (2021). Bacteriophages in water pollution control: advantages and limitations. Front. Environ. Sci. Eng. 15 (5), 84. doi: 10.1007/s11783-020-1378-y 33294248 PMC7716794

[B26] Jiang G. Wei Z. Xu J. Chen H. Zhang Y. She X.. (2017). Bacterial wilt in China: history, current status, and future perspectives. Front. Plant Sci. 8. doi: 10.3389/fpls.2017.01549 PMC5601990 28955350

[B27] Jones J. B. Jackson L. E. Balogh B. Obradovic A. Iriarte F. B. Momol M. T. (2007). Bacteriophages for plant disease control. Annu. Rev. Phytopathol. 45, 245–262. doi: 10.1146/annurev.phyto.45.062806.094411 17386003

[B28] Jurrus E. Engel D. Star K. Monson K. Brandi J. Felberg L. E.. (2018). Improvements to the APBS biomolecular solvation software suite. Protein Sci. 27, 112–128. doi: 10.1002/pro.3280 28836357 PMC5734301

[B29] Katz L. S. Griswold T. Morrison S. S. Caravas J. A. Zhang S. den Bakker H. C.. (2019). Mashtree: a rapid comparison of whole genome sequence files. J. Open Source Softw 4 (44), 1762. doi: 10.21105/joss.01762 PMC9380445 35978566

[B30] Kaur G. Agarwal R. Sharma R. K. (2021). Bacteriophage therapy for critical and high-priority antibiotic-resistant bacteria and phage cocktail-antibiotic formulation perspective. Food Environ. Virol. 13, 433–446. doi: 10.1007/s12560-021-09483-z 34120319

[B31] Kering K. K. Kibii B. J. Wei H. (2019). Biocontrol of phytobacteria with bacteriophage cocktails. Pest Manag Sci. 75, 1775–1781. doi: 10.1002/ps.5324 30624034

[B32] Kropinski A. M. Mazzocco A. Waddell T. E. Lingohr E. Johnson R. P. (2009). Enumeration of bacteriophages by double agar overlay plaque assay. Methods Mol. Biol. 501, 69–76. doi: 10.1007/978-1-60327-164-6_7 19066811

[B33] Letunic I. Bork P. (2021). Interactive Tree Of Life (iTOL) v5: an online tool for phylogenetic tree display and annotation. Nucleic Acids Res. 49, W293–w296. doi: 10.1093/nar/gkab301 33885785 PMC8265157

[B34] Lin Z. Gu G. Chen C. Zhou T. Hu F. Cai X. (2023). Characterization and complete genome sequence analysis of the novel phage RPZH3 infecting *Ralstonia solanacearum* . Arch. Virol. 168, 105. doi: 10.1007/s00705-023-05737-2 36899129

[B35] Lowe-Power T. Avalos J. Bai Y. Munoz M. C. Chipman K. Tom C. E.. (2020). A meta-analysis of the known global distribution and host range of the Ralstonia species complex. bioRxiv. doi: 10.1101/2020.07.13.189936

[B36] Magar R. Lee S. Y. Kim H. J. Lee S. W. (2022). Biocontrol of bacterial wilt in tomato with a cocktail of lytic bacteriophages. Appl. Microbiol. Biotechnol. 106, 3837–3848. doi: 10.1007/s00253-022-11962-7 35562488

[B37] Mansfield J. Genin S. Magori S. Citovsky V. Sriariyanum M. Ronald P.. (2012). Top 10 plant pathogenic bacteria in molecular plant pathology. Mol. Plant Pathol. 13, 614–629. doi: 10.1111/j.1364-3703.2012.00804.x 22672649 PMC6638704

[B38] Markwitz P. Lood C. Olszak T. van Noort V. Lavigne R. Drulis-Kawa Z. (2022). Genome-driven elucidation of phage-host interplay and impact of phage resistance evolution on bacterial fitness. ISME J. 16, 533–542. doi: 10.1038/s41396-021-01096-5 34465897 PMC8776877

[B39] Mourosi J. Awe A. Guo W. Batra H. Ganesh H. Wu X.. (2022). Understanding bacteriophage tail fiber interaction with host surface receptor: the key "blueprint" for reprogramming phage host range. Int. J. Mol. Sci. 23 (20), 12146. doi: 10.3390/ijms232012146 36292999 PMC9603124

[B40] Mushegian A. R. (2020). Are there 10(31) virus particles on earth, or more, or fewer? J. Bacteriol 202 (9), 2–20. doi: 10.1128/JB.00052-20 PMC7148134 32071093

[B41] Nobrega F. L. Vlot M. de Jonge P. A. Dreesens L. L. Beaumont H. J. E. Lavigne R.. (2018). Targeting mechanisms of tailed bacteriophages. Nat. Rev. Microbiol. 16, 760–773. doi: 10.1038/s41579-018-0070-8 30104690

[B42] Ondov B. D. Treangen T. J. Melsted P. Mallonee A. B. Bergman N. H. Koren S.. (2016). Mash: fast genome and metagenome distance estimation using MinHash. Genome Biol. 17, 132. doi: 10.1186/s13059-016-0997-x 27323842 PMC4915045

[B43] Pandit M. A. Kumar J. Gulati S. Bhandari N. Mehta P. Katyal R.. (2022). Major biological cantrol strategies for plant pathogens. Pathogens 11 (2), 273. doi: 10.3390/pathogens11020273 35215215 PMC8879208

[B44] Paudel S. Dobhal S. Alvarez A. M. Arif M. (2020). Taxonomy and phylogenetic research on *Ralstonia solanacearum* species complex: A complex pathogen with extraordinary economic consequences. Pathogens 9 (11), 886. doi: 10.3390/pathogens9110886 33113847 PMC7694096

[B45] Piya D. Vara L. Russell W. K. Young R. Gill J. J. (2017). The multicomponent antirestriction system of phage P1 is linked to capsid morphogenesis. Mol. Microbiol. 105, 399–412. doi: 10.1111/mmi.13705 28509398 PMC6011833

[B46] Procter J. B. Carstairs G. M. Soares B. Mourão K. Ofoegbu T. C. Barton D.. (2021). Alignment of biological sequences with jalview. Methods Mol. Biol. 2231, 203–224. doi: 10.1007/978-1-0716-1036-7_13 33289895 PMC7116599

[B47] Raetz C. R. Whitfield C. (2002). Lipopolysaccharide endotoxins. Annu. Rev. Biochem. 71, 635–700. doi: 10.1016/0167-5699(92)90009-V 12045108 PMC2569852

[B48] Rakhuba D. V. Kolomiets E. I. Dey E. S. Novik G. I. (2010). Bacteriophage receptors, mechanisms of phage adsorption and penetration into host cell. Pol. J. Microbiol. 59, 145–155. doi: 10.33073/pjm- 21033576

[B49] Rosignoli S. Paiardini A. (2022). Boosting the full potential of PyMOL with structural biology plugins. Biomolecules 12 (12), 1764. doi: 10.3390/biom12121764 36551192 PMC9775141

[B50] Shaw J. Yu Y. W. (2023). Fast and robust metagenomic sequence comparison through sparse chaining with skani. Nat. Methods 20, 1661–1665. doi: 10.1038/s41592-023-02018-3 37735570 PMC10630134

[B51] Sievers F. Higgins D. G. (2018). Clustal Omega for making accurate alignments of many protein sequences. Protein Sci. 27, 135–145. doi: 10.1002/pro.3290 28884485 PMC5734385

[B52] Sullivan M. J. Petty N. K. Beatson S. A. (2011). Easyfig: a genome comparison visualizer. Bioinformatics 27, 1009–1010. doi: 10.1093/bioinformatics/btr039 21278367 PMC3065679

[B53] Tang Y. Zhou M. Yang C. Liu R. Du H. Ma M. (2024). Advances in isolated phages that affect *Ralstonia solanacearum* and their application in the biocontrol of bacterial wilt in plants. Lett. Appl. Microbiol. 77 (4), 37. doi: 10.1093/lambio/ovae037 38573829

[B54] Tian Y. Wu L. Lu R. Bao H. Zhou Y. Pang M.. (2022). Virulent phage vB_CpeP_HN02 inhibits clostridium perfringens on the surface of the chicken meat. Int. J. Food Microbiol. 363, 109514. doi: 10.1016/j.ijfoodmicro.2021.109514 34999475

[B55] Trivedi P. Leach J. E. Tringe S. G. Sa T. Singh B. K. (2020). Plant-microbiome interactions: from community assembly to plant health. Nat. Rev. Microbiol. 18 (11), 607–621. doi: 10.1038/s41579-020-0412-1 32788714

[B56] Villalpando-Aguilar J. L. Matos-Pech G. López-Rosas I. Castelán-Sánchez H. G. Alatorre-Cobos F. (2022). Phage therapy for crops: concepts, experimental and bioinformatics approaches to direct its application. Int. J. Mol. Sci. 24 (1), 325. doi: 10.3390/ijms24010325 36613768 PMC9820149

[B57] Vyas (1991). Atomic features of protein-carbohydrate interactions. Curr. Opin. Struct. Biol. 1, 732–740. doi: 10.1016/0959-440X(91)90172-P

[B58] Wang K. Chen D. Liu Q. Zhu P. Sun M. Peng D. (2022). Isolation and characterization of novel lytic bacteriophage vB_RsoP_BMB50 infecting *Ralstonia solanacearum* . Curr. Microbiol. 79, 245. doi: 10.1007/s00284-022-02940-3 35834130

[B59] Wang R. Cong Y. Mi Z. Fan H. Shi T. Liu H.. (2019). Characterization and complete genome sequence analysis of phage GP4, a novel lytic Bcep22-like podovirus. Arch. Virol. 164, 2339–2343. doi: 10.1007/s00705-019-04309-7 31214785

[B60] Wang X. Wang S. Huang M. He Y. Guo S. Yang K.. (2024). Phages enhance both phytopathogen density control and rhizosphere microbiome suppressiveness. mBio 15, e0301623. doi: 10.1128/mbio.03016-23 38780276 PMC11237578

[B61] Wang X. Wei Z. Yang K. Wang J. Jousset A. Xu Y.. (2019). Phage combination therapies for bacterial wilt disease in tomato. Nat. Biotechnol. 37, 1513–1520. doi: 10.1038/s41587-019-0328-3 31792408

[B62] Waterhouse A. Bertoni M. Bienert S. Studer G. Tauriello G. Gumienny R.. (2018). SWISS-MODEL: homology modelling of protein structures and complexes. Nucleic Acids Res. 46, W296–w303. doi: 10.1093/nar/gky427 29788355 PMC6030848

[B63] Wei C. Liu J. Maina A. N. Mwaura F. B. Yu J. Yan C.. (2017). Developing a bacteriophage cocktail for biocontrol of potato bacterial wilt. Virol. Sin. 32, 476–484. doi: 10.1007/s12250-017-3987-6 29168148 PMC6598922

[B64] Wicker E. Grassart L. Coranson-Beaudu R. Mian D. Guilbaud C. Fegan M.. (2007). *Ralstonia solanacearum* strains from Martinique (French West Indies) exhibiting a new pathogenic potential. Appl. Environ. Microbiol. 73, 6790–6801. doi: 10.1128/AEM.00841-07 17720825 PMC2074947

[B65] Wilcox S. A. Toder R. Foster J. W. (1996). Rapid isolation of recombinant lambda phage DNA for use in fluorescence in *situ* hybridization. Chromosome Res. 4, 397–398. doi: 10.1007/BF02257276 8871829

[B66] Ye M. Sun M. Huang D. Zhang Z. Zhang H. Zhang S.. (2019). A review of bacteriophage therapy for pathogenic bacteria inactivation in the soil environment. Environ. Int. 129, 488–496. doi: 10.1016/j.envint.2019.05.062 31158595

[B67] Zhao Q. Geng M. Y. Xia C. J. Lei T. Wang J. Cao C. D.. (2023). Identification, genetic diversity, and pathogenicity of *Ralstonia pseudosolanacearum* causing cigar tobacco bacterial wilt in China. FEMS Microbiol. Ecol. 99 (3), 18. doi: 10.1093/femsec/fiad018 36822630

[B68] Zheng J. Chen W. Xiao H. Yang F. Song J. Cheng L.. (2023). Asymmetric structure of podophage GP4 reveals a novel architecture of three types of tail fibers. J. Mol. Biol. 435, 168258. doi: 10.1016/j.jmb.2023.168258 37660940

