# Corrigendum: Development of a lytic Ralstonia phage cocktail and evaluation of its control efficacy against tobacco bacterial wilt

**DOI:** 10.3389/fpls.2025.1628569

**Published:** 2025-06-10

**Authors:** Haoxin He, Ke Yi, Lei Yang, Yongfeng Jing, Lifu Kang, Zhihao Gao, Dong Xiang, Ge Tan, Yunsheng Wang, Qian Liu, Lin Xie, Shiya Jiang, Tianbo Liu, Wu Chen

**Affiliations:** ^1^ College of Plant Protection, Hunan Agricultural University, Changsha, China; ^2^ Tobacco Leaf Raw Material Procurement Center, China Tobacco Hunan Industrial Co., Ltd, Changsha, China; ^3^ Plant Protection Research Center, Hunan Tobacco Science Research Institute, Changsha, China

**Keywords:** bacteria wilt (BW), *Ralstonia solanacearum*, Ralstonia phage, phage cocktail, control efficacy, tail fiber

In the published article, there was an error in **Figure 1B** as published. The wrong **Figure 1B** was uploaded (the picture of YL5 wrongly overwrote the pictures of YL8 and YL9). The corrected **Figure 1** and its caption “Control efficiency of phages and cocktails against BW in pots.” appear below.

**Figure 1:**
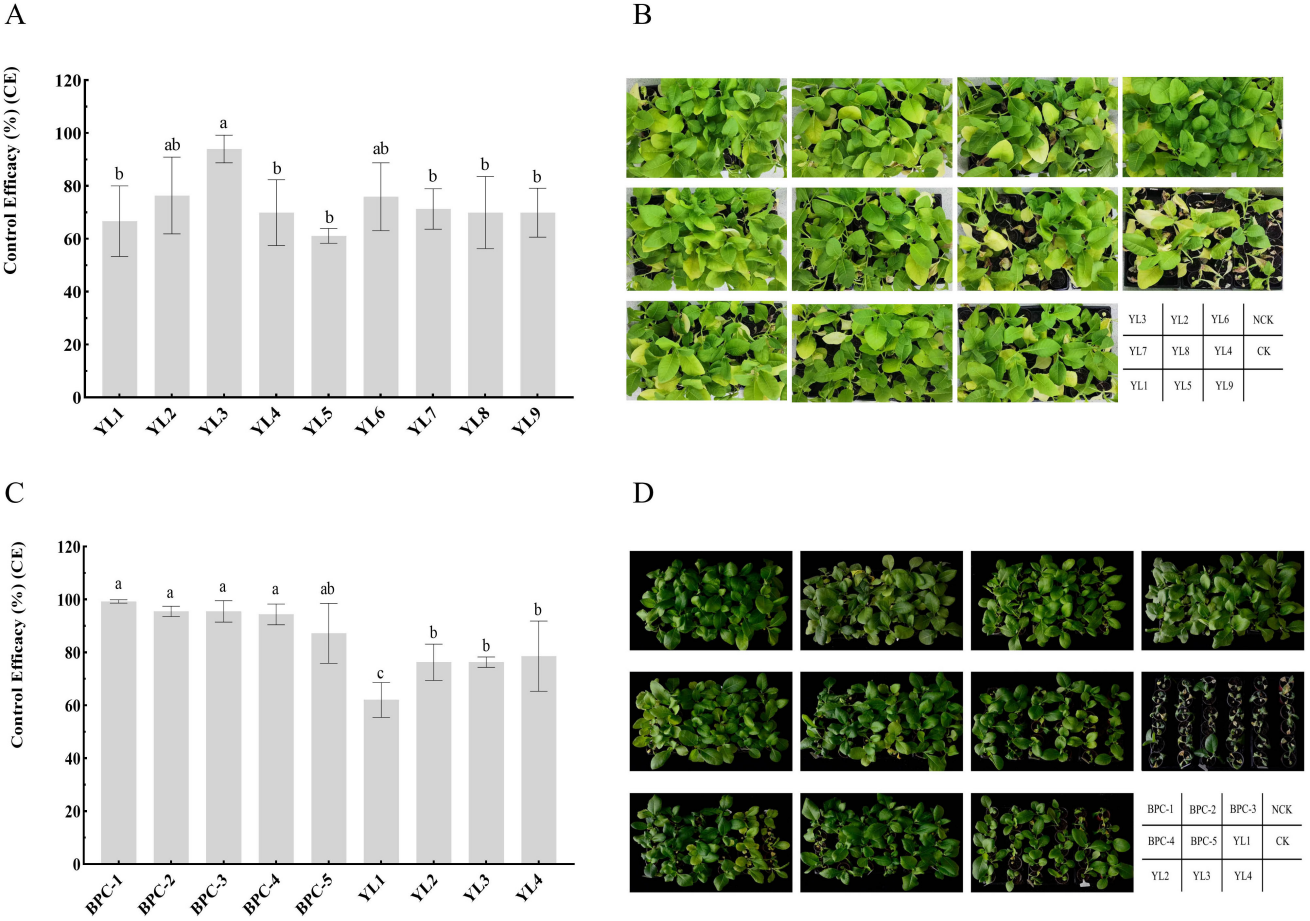
Control efficiency of phages and phage cocktails against BW in pots. **(A, B)** show the evaluation of single-phage biocontrol potential; **(C, D)** show the control efficacy of phage cocktails against tobacco BW inoculation with three *R. pseudosolanacearium* strains. Letters in the bar chart indicate significant differences according to Duncan’s analysis (P ≤ 0.05); NCK is the negative control group.

The authors apologize for this error and state that this does not change the scientific conclusions of the article in any way. The original article has been updated.

