# Supplementary material for: Development of a lytic Ralstonia phage cocktail and evaluation of its control efficacy against tobacco bacterial wilt
Source: Front Plant Sci. 2025 Mar 13;16:1554992. doi: 10.3389/fpls.2025.1554992 (PMC11966396; doi:10.3389/fpls.2025.1554992)
Supplement: Supplementary file 1 [file DataSheet1.docx]

Supplementary Material

# Supplementary Figures and Tables

## Supplementary Figures


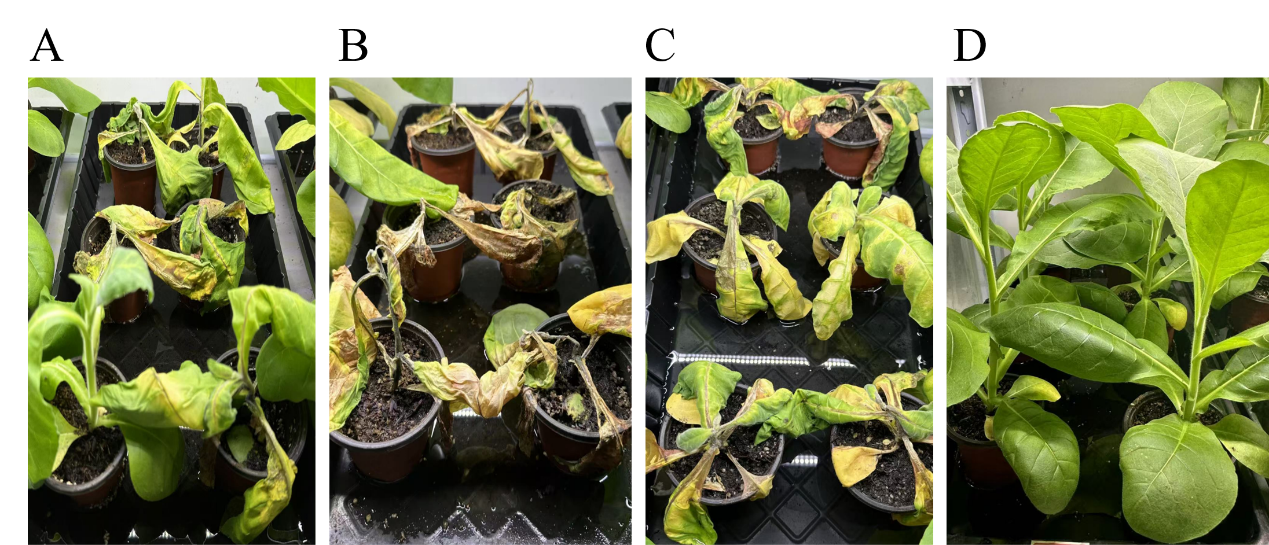
**Figure S1.** Virulence effects of RStab-5, RStab-12, and RStab-19 inoculation on tobacco. Figures S1A, S3B, S3C, and S3D show the effects of RStab-5, RStab-12, RStab-19, and NCK (negative control group), respectively.


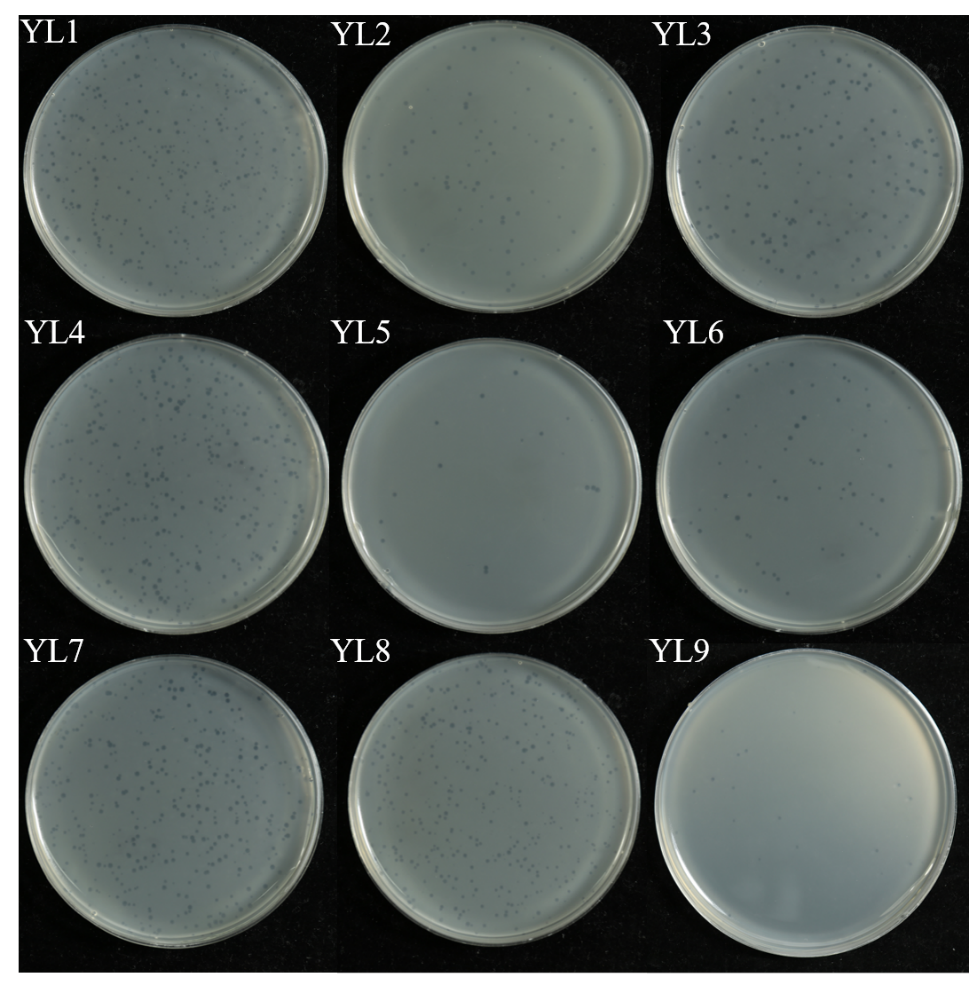


**Figure S2** Plaques formed by the nine phages.
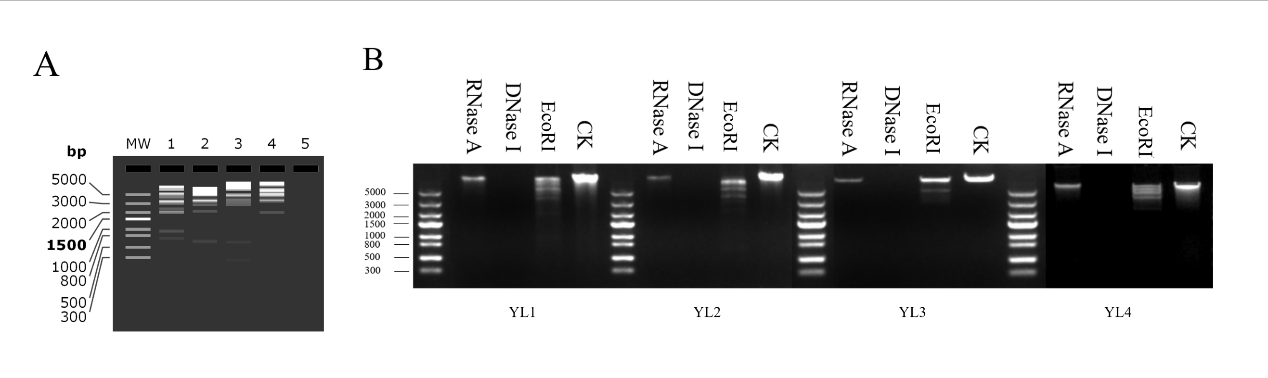


**Figure** **S3** Electrophoresis of four phage strains after nuclease and endonuclease treatment. Figure S4A shows the predicted electrophoretic banding patterns of the genomes of the four phage strains after EcoRI treatment using SnapGene, with lanes 1, 2, 3, and 4 indicating YL1, YL2, YL3, and YL4, respectively. Figure S4B shows the electrophoretic banding patterns of the four phage strains after nuclease and EcoRI treatment, with different phages separated by a marker. The different phages are YL1, YL2, YL3, and YL4 in order from left to right, and each phage treatment had four lanes (RNase A, DNase I, EcoRI, and CK, from left to right).


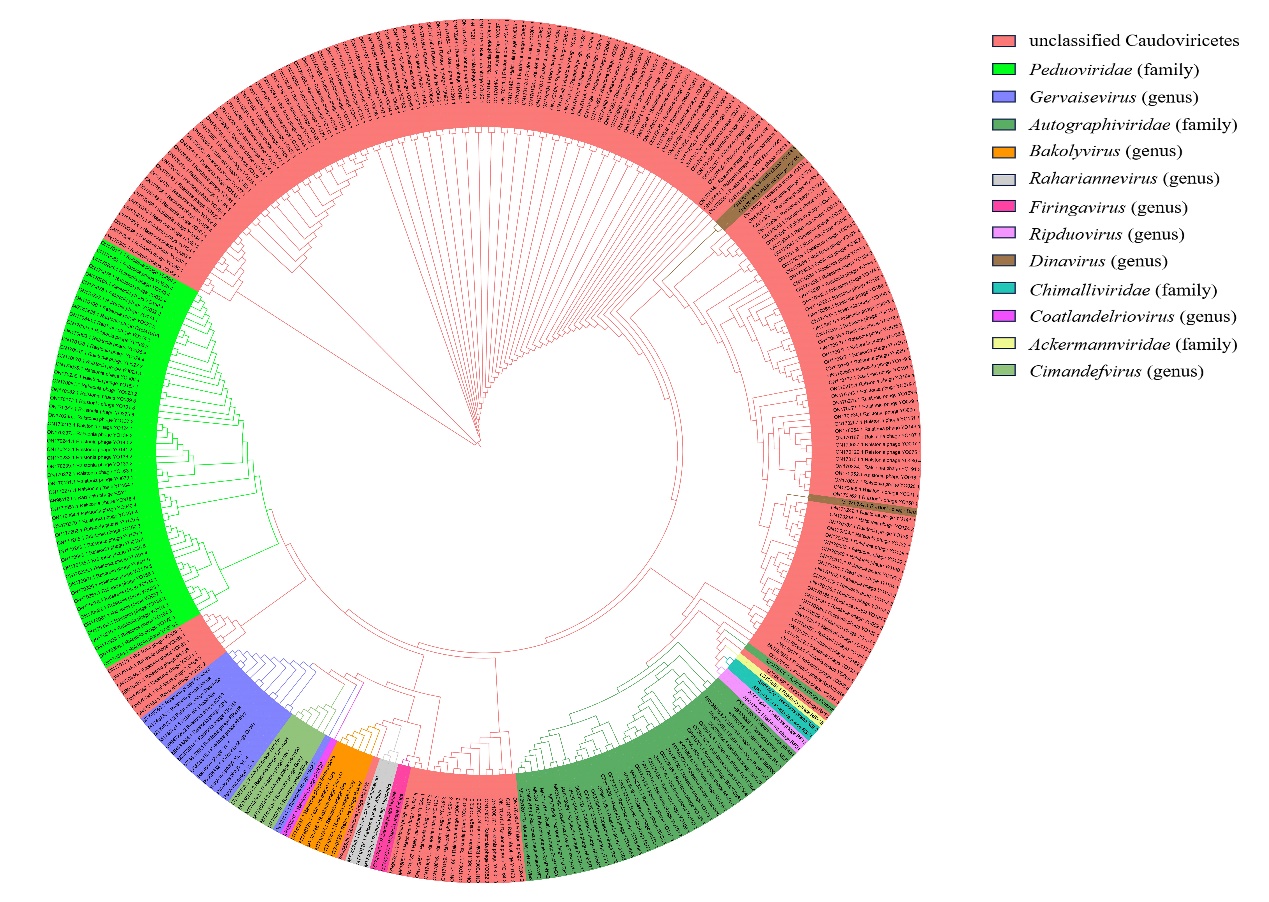


**Figure S4** Phylogenetic analysis of four phages with 317 Ralstonia phages in the Caudoviricetes class. Different colored squares represent different taxonomic relationships among phages.


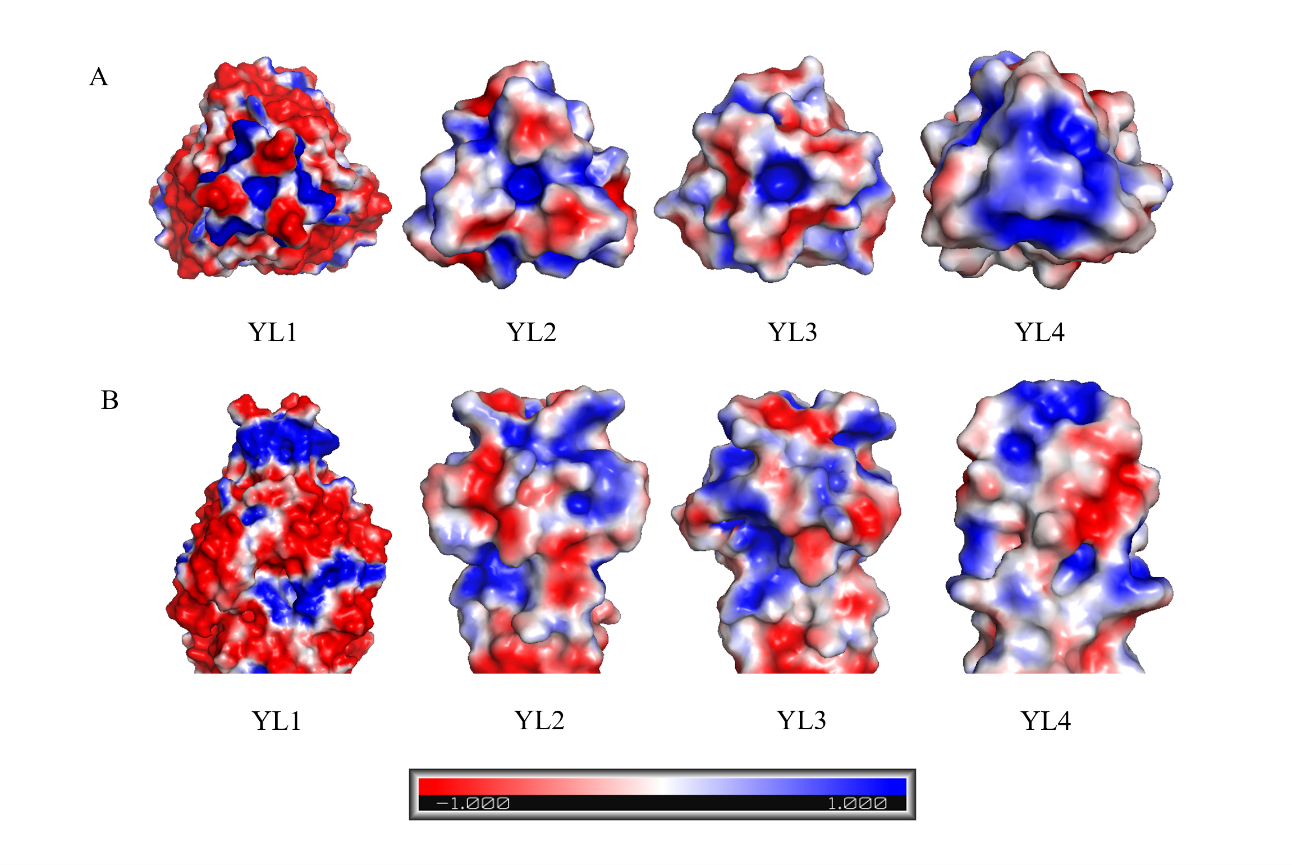


**Figure S5** Surface charge distribution of the “tip domain” of tail fiber proteins from four phages. Figure S6A shows the top view of the “tip domain”. Figure S6B shows the side view of the “tip domain”. From left to right: YL1, YL2, YL3, and YL4. Blue indicates a positive charge, and red indicates a negative charge.

**Table S1** Determination of the phage host range.

| Number | Origin | Isolated location | YL1 | YL2 | YL3 | YL4 | YL5 | YL6 | YL7 | YL8 | YL9 |
| --- | --- | --- | --- | --- | --- | --- | --- | --- | --- | --- | --- |
| RStab-1 | tobacco | Jinshui Village, Daolin Township, Ningxiang City | + | +++ | + | + | ++ | ++ | - | + | ++ |
| RStab-2 | tobacco | Jinshui Village, Daolin Township, Ningxiang City | + | + | + | + | ++ | + | - | + | + |
| RStab-3 | tobacco | Tangshi Community, Old Granary Township, Ningxiang City | - | - | - | + | - | - | - | - | - |
| RStab-4 | tobacco | Erquan Village, Dachengqiao Town, Ningxiang City | +++ | +++ | - | +++ | +++ | - | - | +++ | - |
| RStab-5 | tobacco | Lotus Hill Village, Jiwei Town, Huayuan County | ++ | - | - | ++ | - | - | - | - | - |
| RStab-6 | tobacco | Lotus Hill Village, Jiwei Town, Huayuan County | ++ | + | + | ++ | + | + | - | + | + |
| RStab-7 | tobacco | Lotus Hill Village, Jiwei Town, Huayuan County | ++ | ++ | + | ++ | ++ | ++ | - | ++ | ++ |
| RStab-8 | tobacco | Jiwei Village, Jiwei Town, Huayuan County | +++ | + | - | +++ | - | - | - | +++ | - |
| RStab-9 | tobacco | Jiwei Village, Jiwei Town, Huayuan County | +++ | + | - | +++ | + | - | - | +++ | - |
| RStab-10 | tobacco | Jiwei Village, Jiwei Town, Huayuan County | +++ | + | - | +++ | + | - | - | +++ | - |
| RStab-11 | tobacco | Jiwei Village, Jiwei Town, Huayuan County | +++ | - | - | +++ | + | - | - | +++ | - |
| RStab-12 | tobacco | Dao Er Village, Jiwei Town, Huayuan County | +++ | +++ | +++ | +++ | +++ | +++ | +++ | +++ | +++ |
| RStab-13 | tobacco | Dao Er Village, Jiwei Town, Huayuan County | + | + | + | + | + | + | + | + | + |
| RStab-14 | tobacco | Guandiping Village, Xinglongchang Town, Luxi County | + | + | + | + | + | + | + | + | + |
| RStab-15 | tobacco | Guandiping Village, Xinglongchang Town, Luxi County | +++ | +++ | +++ | +++ | +++ | + | +++ | +++ | +++ |
| RStab-16 | tobacco | Guandiping Village, Xinglongchang Township, Luxi County | ++ | ++ | + | ++ | ++ | ++ | - | ++ | - |
| RStab-17 | tobacco | Jinniu Village, Maliziba Township, Huayuan County | +++ | +++ | +++ | +++ | +++ | ++ | +++ | +++ | +++ |
| RStab-18 | tobacco | Heping Village, Qianling Township, Baoding County | + | - | - | ++ | - | - | - | +++ | - |
| RStab-19 | tobacco | Jiantang Village, Chigongtang Township, Fenghuang County | + | + | - | + | + | + | + | + | + |
| RStab-20 | tobacco | Jiantang Village, Chigongtang Township, Fenghuang County | + | +++ | +++ | + | +++ | +++ | +++ | + | +++ |
| RStab-21 | tobacco | Jiantang Village, Chigongtang Township, Fenghuang County | + | + | + | + | + | + | - | + | + |
| RStab-22 | tobacco | Jiantang Village, Chigongtang Township, Fenghuang County | - | ++ | + | - | + | ++ | + | + | + |
| RStab-23 | tobacco | Xiva Village, Xinglongchang Township, Luxi County | - | - | - | - | - | - | - | - | +++ |
| RStab-24 | tobacco | Xiva Village, Xinglongchang Township, Luxi County | - | - | - | - | - | - | - | - | - |
| RStab-25 | tobacco | Xiva Village, Xinglongchang Township, Luxi County | - | - | - | - | - | - | - | - | - |
| RStab-26 | tobacco | Xiva Village, Xinglongchang Township, Luxi County | - | - | - | - | - | - | - | - | - |
| RStab-27 | tobacco | Hunan Institute of Microbiology, Changsha | +++ | - | + | +++ | - | + | - | +++ | - |
| RStab-28 | tobacco | Hunan Institute of Microbiology, Changsha | +++ | +++ | +++ | +++ | +++ | +++ | +++ | - | +++ |
| RStab-29 | tobacco | Hunan Institute of Microbiology, Changsha | + | +++ | +++ | + | +++ | +++ | +++ | + | +++ |
| RStab-30 | tobacco | Hunan Institute of Microbiology, Changsha | + | +++ | +++ | + | +++ | +++ | +++ | + | +++ |
| RStab-31 | tobacco | Hunan Institute of Microbiology, Changsha | + | +++ | +++ | + | +++ | +++ | +++ | + | +++ |
| RSpep-1 | pepper | Hunan Institute of Microbiology, Changsha | +++ | - | - | +++ | - | - | - | +++ | - |
| RSpep-2 | pepper | Hunan Institute of Microbiology, Changsha | + | - | - | - | - | - | - | + | - |
| RSpot-1 | potato | Biotechnlology Research Center, Shandong Academy of Agricultural Sciences | +++ | +++ | +++ | +++ | +++ | ++ | +++ | +++ | +++ |
| RSpot-2 | potato | Biotechnlology Research Center, Shandong Academy of Agricultural Sciences | + | + | - | - | - | - | - | + | - |
| RSpea-1 | peanut | Biotechnlology Research Center, Shandong Academy of Agricultural Sciences | +++ | +++ | +++ | +++ | +++ | +++ | - | +++ | ++ |
| RSpea-2 | peanut | Biotechnlology Research Center, Shandong Academy of Agricultural Sciences | + | - | - | + | - | - | - | - | - |
| RSpea-3 | peanut | Biotechnlology Research Center, Shandong Academy of Agricultural Sciences | +++ | +++ | +++ | +++ | +++ | +++ | - | +++ | +++ |
| Lysis rates (%) | | | 84.21 | 65.79 | 52.63 | 81.58 | 65.79 | 57.89 | 34.21 | 78.95 | 55.26 |

**Table S2** ORF analysis of the YL1 genome.

| ORF | START | END | STRAND | Length(aa) | Accession | Top BLAST hit | Species | identity(%) | Evalue |
| --- | --- | --- | --- | --- | --- | --- | --- | --- | --- |
| 1 | 1 | 1536 | + | 511 | YP_010078593.1 | virion structural protein | Ralstonia phage Claudette | 99.41 | 5.71e-316 |
| 2 | 1624 | 11130 | + | 3168 | YP_010078592.1 | DarB-like antirestriction | Ralstonia phage Claudette | 95.90 | 0 |
| 3 | 11826 | 11605 | - | 73 | YP_010078200.1 | hypothetical protein | Ralstonia phage Cimandef | 100.00 | 4.55E-49 |
| 4 | 12180 | 11869 | - | 103 | WAX26341.1 | hypothetical protein | Ralstonia phage p2110 | 99.03 | 4.56E-65 |
| 5 | 12344 | 12952 | + | 202 | WAX26317.1 | putative peptidoglycan binding protein | Ralstonia phage p2110 | 99.01 | 4.16E-145 |
| 6 | 12949 | 13176 | + | 75 | YP_010078796.1 | holin | Ralstonia phage GP4 | 100.00 | 7.19E-47 |
| 7 | 13269 | 13649 | + | 126 | YP_010078693.1 | hol-like chemotaxis | Ralstonia phage Gervaise | 100.00 | 2.39E-80 |
| 8 | 13624 | 14028 | + | 134 | YP_010078694.1 | hypothetical protein | Ralstonia phage Gervaise | 99.25 | 1.06E-72 |
| 9 | 14028 | 14561 | + | 177 | WAX26320.1 | hypothetical protein | Ralstonia phage p2110 | 98.87 | 3.09E-112 |
| 10 | 15712 | 14558 | - | 384 | WAX26350.1 | integrase | Ralstonia phage p2110 | 95.82 | 3.11E-267 |
| 11 | 16159 | 16698 | + | 179 | YP_010078697.1 | hypothetical protein | Ralstonia phage Gervaise | 97.21 | 3.94E-125 |
| 12 | 16947 | 16681 | - | 88 | UAW01014.1 | pyocin activator protein | Ralstonia phage RPZH3 | 97.73 | 1.27E-55 |
| 13 | 18209 | 16959 | - | 416 | YP_010078788.1 | hypothetical protein | Ralstonia phage GP4 | 72.66 | 1.26E-159 |
| 14 | 19297 | 18233 | - | 354 | YP_010078700.1 | DNA polymerase processivity factor | Ralstonia phage Gervaise | 79.10 | 2.34E-197 |
| 15 | 19791 | 19312 | - | 159 | YP_010078786.1 | endonuclease | Ralstonia phage GP4 | 88.05 | 2.64E-102 |
| 16 | 19990 | 19784 | - | 68 | YP_010078785.1 | DNA-binding protein | Ralstonia phage GP4 | 89.71 | 2.40E-38 |
| 17 | 20922 | 19996 | - | 308 | WAX26347.1 | hypothetical protein | Ralstonia phage p2110 | 97.40 | 3.92E-212 |
| 18 | 21359 | 20937 | - | 140 | YP_009638224.1 | endonuclease | Mycobacterium phage Jeffabunny | 42.71 | 3.22E-20 |
| 19 | 22362 | 21388 | - | 324 | YP_010078325.1 | RecE-like recombination exonuclease | Ralstonia phage Eline | 96.91 | 1.47E-224 |
| 20 | 22609 | 23085 | + | 158 | YP_010078782.1 | Fis family transcriptional regulator | Ralstonia phage GP4 | 99.37 | 8.57E-112 |
| 21 | 23691 | 23101 | - | 196 | WAX26352.1 | hypothetical protein | Ralstonia phage p2110 | 99.49 | 1.06E-135 |
| 22 | 23820 | 24188 | + | 122 | QKW95382.1 | transcriptional regulator | Ralstonia phage RPZH6 | 100.00 | 3.87E-75 |
| 23 | 24348 | 24920 | + | 190 | YP_010078648.1 | plasmid-derived single-stranded DNA-binding protein | Ralstonia phage Claudette | 98.95 | 4.89E-129 |
| 24 | 24929 | 25153 | + | 74 | WAX26324.1 | hypothetical protein | Ralstonia phage p2110 | 87.67 | 6.83E-40 |
| 25 | 25140 | 25625 | + | 161 | QKW95377.1 | hypothetical protein | Ralstonia phage RPZH6 | 88.27 | 5.85E-96 |
| 26 | 25625 | 26011 | + | 128 | WAX26326.1 | hypothetical protein | Ralstonia phage p2110 | 93.75 | 1.32E-87 |
| 27 | 26193 | 26936 | + | 247 | WAX26327.1 | hypothetical protein | Ralstonia phage p2110 | 100.00 | 4.24E-174 |
| 28 | 26933 | 27367 | + | 144 | YP_010078219.1 | hypothetical protein | Ralstonia phage Cimandef | 88.41 | 1.01E-82 |
| 29 | 27962 | 28840 | + | 292 | QKW95373.1 | hypothetical protein | Ralstonia phage RPZH6 | 84.59 | 8.08E-174 |
| 30 | 28840 | 29697 | + | 285 | QKW95372.1 | replication protein | Ralstonia phage RPZH6 | 65.08 | 7.04E-94 |
| 31 | 29697 | 30503 | + | 268 | QKW95371.1 | DnaC-like protein | Ralstonia phage RPZH6 | 98.88 | 1.22E-188 |
| 32 | 30586 | 31041 | + | 151 | YP_010078450.1 | hypothetical protein | Ralstonia phage Gerry | 74.19 | 2.85E-25 |
| 33 | 31069 | 31326 | + | 85 | QKW95367.1 | hypothetical protein | Ralstonia phage RPZH6 | 95.29 | 4.14E-53 |
| 34 | 31323 | 31793 | + | 156 | YP_010078226.1 | hypothetical protein | Ralstonia phage Cimandef | 94.19 | 1.48E-102 |
| 35 | 32748 | 32320 | - | 142 | YP_010078277.1 | hypothetical protein | Ralstonia phage Eline | 90.85 | 1.27E-89 |
| 36 | 33106 | 33744 | + | 212 | YP_010078629.1 | terminase small subunit | Ralstonia phage Claudette | 95.28 | 1.86E-137 |
| 37 | 33900 | 34094 | + | 64 | no hit | hypothetical protein | no hit |  |  |
| 38 | 34123 | 34431 | + | 102 | YP_010078755.1 | hypothetical protein | Ralstonia phage GP4 | 84.40 | 4.00E-51 |
| 39 | 34485 | 34643 | + | 52 | YP_010078754.1 | hypothetical protein | Ralstonia phage GP4 | 98.08 | 1.01E-23 |
| 40 | 34640 | 34783 | + | 47 | YP_010078753.1 | hypothetical protein | Ralstonia phage GP4 | 97.87 | 1.92E-21 |
| 41 | 34780 | 35130 | + | 116 | YP_008853822.1 | hypothetical protein | Ralstonia phage RSK1 | 94.83 | 1.62E-71 |
| 42 | 35142 | 35363 | + | 73 | YP_010078729.1 | hypothetical protein | Ralstonia phage Gervaise | 97.26 | 5.81E-43 |
| 43 | 35389 | 36048 | + | 219 | WAX26285.1 | hypothetical protein | Ralstonia phage p2110 | 95.85 | 2.01E-148 |
| 44 | 36045 | 36932 | + | 295 | no hit | hypothetical protein | no hit |  |  |
| 45 | 36929 | 37159 | + | 76 | YP_010078519.1 | hypothetical protein | Ralstonia phage Heva | 95.95 | 4.45E-41 |
| 46 | 37159 | 37350 | + | 63 | YP_010078616.1 | hypothetical protein | Ralstonia phage Claudette | 93.65 | 2.50E-35 |
| 47 | 37418 | 37864 | + | 148 | DAO82797.1 | MAG TPA: putative proteolytic subunit | Siphoviridae sp. | 38.41 | 1.03E-14 |
| 48 | 37857 | 39398 | + | 513 | QMV32797.1 | terminase large subunit | Ralstonia phage Darius | 96.10 | 0 |
| 49 | 39395 | 39820 | + | 141 | WAX26293.1 | hypothetical protein | Ralstonia phage p2110 | 96.45 | 1.27E-98 |
| 50 | 39836 | 42181 | + | 781 | QKW95341.1 | portal protein | Ralstonia phage RPZH6 | 99.49 | 0 |
| 51 | 42192 | 43142 | + | 316 | QKW95340.1 | hypothetical protein | Ralstonia phage RPZH6 | 89.87 | 3.49E-149 |
| 52 | 43168 | 43428 | + | 86 | YP_010078737.1 | hypothetical protein | Ralstonia phage GP4 | 98.84 | 9.69E-60 |
| 53 | 43455 | 43652 | + | 65 | YP_010078299.1 | CsrA-like regulator | Ralstonia phage Eline | 95.38 | 3.37E-34 |
| 54 | 44281 | 43649 | - | 210 | YP_010078300.1 | HNH endonuclease | Ralstonia phage Eline | 78.88 | 2.45E-91 |
| 55 | 44333 | 44785 | + | 150 | QKW95336.1 | AP2 domain-containing protein | Ralstonia phage RPZH6 | 72.19 | 2.49E-73 |
| 56 | 44884 | 45993 | + | 369 | YP_010078301.1 | major head protein | Ralstonia phage Eline | 99.46 | 1.06E-267 |
| 57 | 46071 | 46544 | + | 157 | UAW00953.1 | virion associated protein | Ralstonia phage RPZH3 | 99.36 | 3.92E-103 |
| 58 | 46595 | 47140 | + | 181 | QKW95419.1 | hypothetical protein | Ralstonia phage RPZH6 | 97.24 | 6.00E-121 |
| 59 | 47189 | 47806 | + | 205 | YP_010078814.1 | structural protein | Ralstonia phage GP4 | 97.07 | 1.03E-134 |
| 60 | 47803 | 48423 | + | 206 | YP_010078252.1 | virion structural protein | Ralstonia phage Cimandef | 99.51 | 1.50E-149 |
| 61 | 48433 | 48855 | + | 140 | YP_010078604.1 | head protein | Ralstonia phage Claudette | 99.29 | 8.94E-94 |
| 62 | 48862 | 49839 | + | 325 | WAX26305.1 | hypothetical protein | Ralstonia phage p2110 | 94.43 | 2.94E-153 |
| 63 | 49848 | 51542 | + | 564 | YP_010078362.1 | hypothetical protein | Ralstonia phage Gamede | 95.21 | 7.00E-299 |
| 64 | 51542 | 51823 | + | 93 | YP_010078309.1 | hypothetical protein | Ralstonia phage Eline | 100.00 | 4.11E-61 |
| 65 | 51823 | 53142 | + | 439 | YP_010078310.1 | tail fiber protein Ⅰ | Ralstonia phage Eline | 99.77 | 1.70E-304 |
| 66 | 53228 | 54244 | + | 338 | ULR75131.1 | tail fiber protein Ⅱ | Burkholderia phage JC1 | 54.84 | 2.08E-116 |
| 67 | 54246 | 54944 | + | 232 | YP_010671599.1 | hypothetical protein | Pseudomonas phage Itty13 | 48.48 | 6.48E-15 |
| 68 | 54991 | 56724 | + | 577 | QMV32780.1 | head closure protein 3 | Ralstonia phage Darius | 98.44 | 0 |
| 69 | 56724 | 57044 | + | 106 | YP_010078597.1 | hypothetical protein | Ralstonia phage Claudette | 99.06 | 2.81E-65 |
| 70 | 57120 | 57551 | + | 143 | YP_010078419.1 | acyl-CoA N-acyltransferase | Ralstonia phage Gerry | 98.55 | 1.11E-98 |
| 71 | 57818 | 58045 | + | 75 | QKW95407.1 | acyl-CoA N-acyltransferase | Ralstonia phage RPZH6 | 98.67 | 1.18E-44 |
| 72 | 58035 | 58868 | + | 277 | YP_010078595.1 | virion structural protein | Ralstonia phage Claudette | 99.28 | 5.07E-181 |
| 73 | 59589 | 58855 | - | 244 | no hit | hypothetical protein | no hit |  |  |

**Table S3** ORF analysis of the YL2 genome.

| ORF | START | END | STRAND | Length(aa) | Accession | Top BLAST hit | Species | identity(%) | Evalue |
| --- | --- | --- | --- | --- | --- | --- | --- | --- | --- |
| 1 | 1 | 1536 | + | 511 | YP_010078263.1 | virion structural protein | Ralstonia phage Cimandef | 90.66 | 6.65E-286 |
| 2 | 1625 | 14926 | + | 4433 | YP_010078688.1 | DarB-like antirestriction | Ralstonia phage Gervaise | 91.87 | 0 |
| 3 | 15224 | 14904 | - | 106 | WAX26341.1 | hypothetical protein | Ralstonia phage p2110 | 97.92 | 3.93E-60 |
| 4 | 15388 | 15996 | + | 202 | YP_010078318.1 | endolysin | Ralstonia phage Eline | 95.05 | 1.68E-135 |
| 5 | 15388 | 15996 | + | 75 | YP_010078796.1 | holin | Ralstonia phage GP4 | 100.00 | 7.19E-47 |
| 6 | 16313 | 16693 | + | 126 | YP_010078693.1 | hol-like chemotaxis | Ralstonia phage Gervaise | 100.00 | 2.39E-80 |
| 7 | 16668 | 17072 | + | 134 | YP_010078657.1 | hypothetical protein | Ralstonia phage Claudette | 91.04 | 1.83E-73 |
| 8 | 17069 | 17605 | + | 178 | YP_010078206.1 | Rz-like spanin | Ralstonia phage Cimandef | 94.94 | 3.58E-107 |
| 9 | 17679 | 17855 | + | 58 | YP_010078792.1 | hypothetical protein | [Ralstonia phage GP4 | 100.00 | 3.40E-30 |
| 10 | 19047 | 17845 | - | 400 | YP_010078791.1 | tyrosine recombinase | Ralstonia phage GP4 | 91.50 | 4.16E-267 |
| 11 | 19611 | 19324 | - | 95 | CAH0532160.1 | hypothetical protein | Ralstonia phage UAM5 | 92.47 | 1.63E-54 |
| 12 | 20930 | 19617 | - | 437 | WAX26344.1 | hypothetical protein | Ralstonia phage p2110 | 89.82 | 9.24E-228 |
| 13 | 22027 | 20963 | - | 354 | WAX26345.1 | hypothetical protein | Ralstonia phage p2110 | 98.02 | 7.74E-242 |
| 14 | 22279 | 22052 | - | 75 | QKW95387.1 | DNA-binding protein | Ralstonia phage RPZH6 | 98.67 | 7.19E-47 |
| 15 | 23241 | 22285 | - | 318 | YP_010078784.1 | RecT-like ssDNA annealing protein | Ralstonia phage GP4 | 100.00 | 8.83E-226 |
| 16 | 24308 | 23280 | - | 342 | YP_010078783.1 | RecE-like recombination exonuclease | Ralstonia phage GP4 | 95.91 | 4.75E-230 |
| 17 | 24492 | 24331 | - |  | no hit | hypothetical protein | no hit |  |  |
| 18 | 24501 | 24977 | + | 158 | YP_010078782.1 | Fis family transcriptional regulator | Ralstonia phage GP4 | 100.00 | 1.04E-112 |
| 19 | 25454 | 24993 | - | 153 | YP_010078781.1 | transcriptional regulator | Ralstonia phage GP4 | 100.00 | 1.86E-106 |
| 20 | 25778 | 26080 | + | 100 | QKW95382.1 | transcriptional regulator | Ralstonia phage RPZH6 | 100.00 | 2.29E-59 |
| 21 | 26240 | 26812 | + | 190 | YP_010078648.1 | plasmid-derived single-stranded DNA-binding protein | Ralstonia phage Claudette | 100.00 | 5.95E-130 |
| 22 | 26821 | 27042 | + | 73 | WAX26324.1 | hypothetical protein | Ralstonia phage p2110 | 100.00 | 3.09E-47 |
| 23 | 27056 | 27523 | + | 155 | WAX26325.1 | hypothetical protein | Ralstonia phage p2110 | 95.48 | 1.60E-101 |
| 24 | 27544 | 27909 | + | 121 | WAX26326.1 | hypothetical protein | Ralstonia phage p2110 | 96.69 | 2.33E-86 |
| 25 | 28093 | 28716 | + | 207 | YP_010078772.1 | GIY-YIG nuclease family protein | Ralstonia phage GP4 | 97.58 | 2.20E-138 |
| 26 | 28713 | 28877 | + | 54 | YP_010078391.1 | hypothetical protein | Ralstonia phage Gamede | 98.15 | 5.16E-31 |
| 27 | 28880 | 29644 | + | 254 | YP_010078392.1 | anti-repressor Ant | Ralstonia phage Gamede | 98.43 | 2.65E-176 |
| 28 | 29641 | 30093 | + | 150 | QKW95374.1 | hypothetical protein | Ralstonia phage RPZH6 | 88.00 | 5.21E-91 |
| 29 | 30381 | 30617 | + | 78 | YP_010078447.1 | hypothetical protein | Ralstonia phage Gerry | 98.72 | 2.55E-46 |
| 30 | 30617 | 31570 | + | 317 | WAX26331.1 | hypothetical protein | Ralstonia phage p2110 | 99.68 | 1.30E-219 |
| 31 | 31557 | 32213 | + | 218 | WAX26332.1 | hypothetical protein | Ralstonia phage p2110 | 98.17 | 2.35E-143 |
| 32 | 32296 | 32715 | + | 139 | QKW95370.1 | hypothetical protein | Ralstonia phage RPZH6 | 81.48 | 8.19E-57 |
| 33 | 32702 | 33046 | + | 114 | YP_010078273.1 | hypothetical protein | Ralstonia phage Eline | 92.11 | 1.63E-70 |
| 34 | 33074 | 33331 | + | 85 | QKW95367.1 | hypothetical protein | Ralstonia phage RPZH6 | 92.94 | 4.84E-52 |
| 35 | 33316 | 33798 | + | 160 | YP_010078226.1 | hypothetical protein | Ralstonia phage Cimandef | 95.48 | 1.23E-102 |
| 36 | 34907 | 34479 | - | 142 | YP_010078277.1 | hypothetical protein | Ralstonia phage Eline | 90.14 | 1.49E-88 |
| 37 | 34479 | 34907 | + | 213 | YP_010078629.1 | terminase small subunit | Ralstonia phage Claudette | 88.21 | 1.34E-128 |
| 38 | 36294 | 36617 | + | 107 | WAX26280.1 | hypothetical protein | Ralstonia phage p2110 | 96.26 | 1.26E-62 |
| 39 | 36668 | 36826 | + | 52 | YP_010078754.1 | hypothetical protein | Ralstonia phage GP4 | 96.15 | 4.12E-23 |
| 40 | 36823 | 36972 | + | 49 | YP_010078341.1 | hypothetical protein | Ralstonia phage Gamede | 63.04 | 2.50E-11 |
| 41 | 36969 | 37319 | + | 116 | YP_008853822.1 | hypothetical protein | Ralstonia phage RSK1 | 93.97 | 1.14E-71 |
| 42 | 37316 | 37552 | + | 78 | YP_010078750.1 | transcriptional regulator | Ralstonia phage GP4 | 93.59 | 4.97E-44 |
| 43 | 37527 | 38153 | + | 208 | WAX26285.1 | hypothetical protein | Ralstonia phage p2110 | 78.90 | 3.62E-123 |
| 44 | 38150 | 38920 | + | 256 | no hit | hypothetical protein | no hit |  |  |
| 45 | 38913 | 39425 | + | 170 | YP_010078186.1 | hypothetical protein | Ralstonia phage Raharianne | 69.79 | 1.06E-36 |
| 46 | 39708 | 39899 | + | 63 | QKW95349.1 | hypothetical protein | Ralstonia phage RPZH6 | 100.00 | 1.65E-27 |
| 47 | 39902 | 40132 | + | 76 | YP_010078744.1 | hypothetical protein | Ralstonia phage GP4 | 95.77 | 9.28E-41 |
| 48 | 40132 | 40323 | + | 63 | QMV32799.1 | hypothetical protein | Ralstonia phage Darius | 96.83 | 7.71E-39 |
| 49 | 40393 | 41964 | + | 523 | CAH0532131.1 | Phage terminase large subunit | Ralstonia phage UAM5 | 87.95 | 0 |
| 50 | 41961 | 42386 | + | 141 | YP_010078741.1 | DNA methyltransferase | Ralstonia phage GP4 | 99.29 | 7.93E-102 |
| 51 | 42403 | 44748 | + | 781 | WAX26294.1 | hypothetical protein | Ralstonia phage p2110 | 98.59 | 0 |
| 52 | 44759 | 45709 | + | 316 | YP_010078244.1 | hypothetical protein | Ralstonia phage Cimandef | 93.06 | 4.67E-148 |
| 53 | 45723 | 45920 | + | 65 | YP_010078246.1 | CsrA-like regulator | Ralstonia phage Cimandef | 100.00 | 1.42E-35 |
| 54 | 45996 | 46292 | + | 98 | YP_010078735.1 | hypothetical protein | Ralstonia phage GP4 | 94.74 | 1.96E-54 |
| 55 | 45996 | 46292 | + | 155 | YP_010078406.1 | hypothetical protein | Ralstonia phage Gerry | 82.69 | 4.37E-90 |
| 56 | 46920 | 48029 | + | 369 | YP_010078301.1 | major head protein | Ralstonia phage Eline | 99.19 | 1.51E-267 |
| 57 | 48107 | 48580 | + | 157 | WAX26300.1 | hypothetical protein | Ralstonia phage p2110 | 99.36 | 4.77E-104 |
| 58 | 48634 | 49173 | + | 179 | YP_010078303.1 | hypothetical protein | Ralstonia phage Eline | 98.88 | 1.80E-121 |
| 59 | 49177 | 49839 | + | 220 | YP_010078814.1 | structural protein | Ralstonia phage GP4 | 98.18 | 1.21E-146 |
| 60 | 49836 | 50456 | + | 206 | YP_010078605.1 | virion structural protein | Ralstonia phage Claudette | 99.51 | 1.06E-149 |
| 61 | 50466 | 50888 | + | 140 | YP_010078306.1 | head protein | Ralstonia phage Gamede | 97.14 | 8.60E-92 |
| 62 | 50895 | 51872 | + | 325 | WAX26305.1 | hypothetical protein | Ralstonia phage p2110 | 98.46 | 1.40E-165 |
| 63 | 51869 | 53575 | + | 568 | WAX26306.1 | hypothetical protein | Ralstonia phage p2110 | 95.77 | 1.28E-302 |
| 64 | 53575 | 53856 | + | 93 | YP_010078309.1 | hypothetical protein | Ralstonia phage Eline | 100.00 | 4.11E-61 |
| 65 | 53856 | 55175 | + | 439 | YP_010078600.1 | tail fiber protein Ⅰ | Ralstonia phage Claudette | 91.12 | 7.06E-281 |
| 66 | 55261 | 56148 | + | 295 | YP_010078807.1 | tail fiber protein Ⅱ | Ralstonia phage GP4 | 92.54 | 1.16E-149 |
| 67 | 56160 | 57893 | + | 577 | QMV32869.1 | head closure protein 3 | Ralstonia phage Dimitile | 96.01 | 0 |
| 68 | 57893 | 58213 | + | 106 | YP_010078805.1 | hypothetical protein | Ralstonia phage GP4 | 99.06 | 1.39E-65 |
| 69 | 58289 | 58720 | + | 143 | YP_010078419.1 | acyl-CoA N-acyltransferase | Ralstonia phage Gerry | 92.75 | 1.70E-93 |
| 70 | 58987 | 59214 | + | 75 | QKW95407.1 | acyl-CoA N-acyltransferase | Ralstonia phage RPZH6 | 100.00 | 2.92E-45 |
| 71 | 59204 | 60037 | + | 277 | YP_010078595.1 | virion structural protein | Ralstonia phage Claudette | 94.95 | 1.22E-172 |
| 72 | 60296 | 60024 | - | 90 | no hit | hypothetical protein | no hit |  |  |
| 73 | 60759 | 60283 | - | 158 | no hit | hypothetical protein | no hit |  |  |

**Table S4** ORF analysis of the YL3 genome.

| ORF | START | END | STRAND | Length(aa) | Accession | Top BLAST hit | Species | identity(%) | Evalue |
| --- | --- | --- | --- | --- | --- | --- | --- | --- | --- |
| 1 | 1 | 1530 | + | 509 | YP_010078593.1 | virion structural protein | Ralstonia phage Claudette | 99.02 | 8.95e-315 |
| 2 | 1588 | 14928 | + | 4446 | YP_010078688.1 | DarB-like antirestriction | Ralstonia phage Gervaise | 94.18 | 0 |
| 3 | 15226 | 14906 | - | 106 | WAX26341.1 | hypothetical protein | Ralstonia phage p2110 | 97.92 | 3.93E-60 |
| 4 | 15390 | 15998 | + | 202 | YP_010078318.1 | endolysin | Ralstonia phage Eline | 96.04 | 1.23E-137 |
| 5 | 15995 | 16222 | + | 75 | YP_010078796.1 | holin | Ralstonia phage GP4 | 100.00 | 7.19E-47 |
| 6 | 16315 | 16695 | + | 126 | YP_010078693.1 | hol-like chemotaxis | Ralstonia phage Gervaise | 100.00 | 2.39E-80 |
| 7 | 16670 | 17074 | + | 134 | YP_010078694.1 | hypothetical protein | Ralstonia phage Gervaise | 100.00 | 2.61E-73 |
| 8 | 17071 | 17607 | + | 178 | YP_010078206.1 | Rz-like spanin | Ralstonia phage Cimandef | 95.51 | 4.35E-108 |
| 9 | 17654 | 17857 | + | 67 | YP_010078792.1 | hypothetical protein | Ralstonia phage GP4 | 98.48 | 1.59E-35 |
| 10 | 19049 | 17847 | - | 400 | YP_010078791.1 | tyrosine recombinase | Ralstonia phage GP4 | 92.25 | 1.77E-268 |
| 11 | 19326 | 19326 | - | 86 | CAH0532160.1 | hypothetical protein | Ralstonia phage UAM5 | 93.02 | 7.31E-51 |
| 12 | 20932 | 19619 | - | 437 | WAX26344.1 | hypothetical protein | Ralstonia phage p2110 | 87.10 | 8.72E-219 |
| 13 | 22025 | 20961 | - | 354 | QKW95389.1 | DNA polymerase III subunit beta | Ralstonia phage RPZH6 | 96.33 | 1.18E-236 |
| 14 | 22277 | 22050 | - | 75 | WAX26346.1 | hypothetical protein | Ralstonia phage p2110 | 96.00 | 4.88E-45 |
| 15 | 23209 | 22283 | - | 308 | YP_010078488.1 | RecT-like ssDNA annealing protein | Ralstonia phage Heva | 96.75 | 1.59E-211 |
| 16 | 24237 | 23278 | - | 319 | YP_010078325.1 | RecE-like recombination exonuclease | Ralstonia phage Eline | 97.49 | 2.32E-222 |
| 17 | 24497 | 24973 | + | 158 | CAH0532169.1 | Phage HNH homing endonuclease | Ralstonia phage UAM5 | 88.12 | 2.67E-60 |
| 18 | 25667 | 24975 | - | 230 | QKW95383.1 | transcriptional regulator | Ralstonia phage RPZH6 | 92.17 | 1.20E-151 |
| 19 | 25760 | 26062 | + | 100 | QKW95382.1 | transcriptional regulator | Ralstonia phage RPZH6 | 100.00 | 2.29E-59 |
| 20 | 26222 | 26794 | + | 190 | YP_010078648.1 | plasmid-derived single-stranded DNA-binding protein | Ralstonia phage Claudette | 100.00 | 5.95E-130 |
| 21 | 26801 | 27022 | + | 73 | WAX26324.1 | hypothetical protein | Ralstonia phage p2110 | 100.00 | 3.09E-47 |
| 22 | 27012 | 27497 | + | 161 | YP_010078774.1 | hypothetical protein | Ralstonia phage GP4 | 85.98 | 5.37E-92 |
| 23 | 27497 | 27877 | + | 126 | CAH0532175.1 | hypothetical protein | Ralstonia phage UAM5 | 86.29 | 2.93E-76 |
| 24 | 28060 | 28683 | + | 207 | YP_010078772.1 | GIY-YIG nuclease family protein | Ralstonia phage GP4 | 99.03 | 5.62E-141 |
| 25 | 28680 | 28844 | + | 54 | YP_010078391.1 | hypothetical protein | Ralstonia phage Gamede | 98.15 | 5.16E-31 |
| 26 | 28847 | 29611 | + | 254 | YP_010078392.1 | anti-repressor Ant | Ralstonia phage Gamede | 94.09 | 1.35E-169 |
| 27 | 29608 | 30060 | + | 150 | QKW95374.1 | hypothetical protein | Ralstonia phage RPZH6 | 88.00 | 5.21E-91 |
| 28 | 30321 | 30584 | + | 87 | YP_010078447.1 | hypothetical protein | Ralstonia phage Gerry | 98.72 | 3.71E-46 |
| 29 | 31109 | 31537 | + | 142 | YP_010078767.1 | hypothetical protein | Ralstonia phage GP4 | 99.30 | 3.20E-96 |
| 30 | 31524 | 32180 | + | 218 | WAX26332.1 | hypothetical protein | Ralstonia phage p2110 | 98.17 | 2.35E-143 |
| 31 | 32263 | 32667 | + | 134 | WAX26333.1 | hypothetical protein | Ralstonia phage p2110 | 81.48 | 4.74E-61 |
| 32 | 32664 | 32978 | + | 104 | CAB4154600.1 | hypothetical protein | uncultured Caudovirales phage | 62.90 | 1.10E-20 |
| 33 | 32965 | 33312 | + | 115 | YP_010078718.1 | terminase small subunit | Ralstonia phage Gervaise | 90.60 | 1.25E-64 |
| 34 | 33338 | 33592 | + | 84 | YP_010078225.1 | hypothetical protein | Ralstonia phage Cimandef | 98.81 | 1.30E-53 |
| 35 | 33577 | 34059 | + | 160 | YP_010078226.1 | hypothetical protein | Ralstonia phage Cimandef | 93.55 | 2.04E-101 |
| 36 | 35006 | 34491 | - | 171 | YP_010078759.1 | hypothetical protein | Ralstonia phage GP4 | 95.68 | 1.96E-110 |
| 37 | 35273 | 35926 | + | 217 | UAW00984.1 | terminase small subunit | Ralstonia phage RPZH3 | 87.10 | 4.98E-126 |
| 38 | 36082 | 36276 | + | 64 | no hit | hypothetical protein | no hit |  |  |
| 39 | 36311 | 36613 | + | 100 | YP_010078755.1 | hypothetical protein | Ralstonia phage GP4 | 84.11 | 2.05E-48 |
| 40 | 36667 | 36825 | + | 52 | YP_010078754.1 | hypothetical protein | Ralstonia phage GP4 | 98.08 | 1.01E-23 |
| 41 | 36822 | 36965 | + | 47 | YP_010078753.1 | hypothetical protein | Ralstonia phage GP4 | 97.87 | 1.92E-21 |
| 42 | 36962 | 37312 | + | 116 | YP_008853822.1 | hypothetical protein | Ralstonia phage RSK1 | 94.83 | 1.62E-71 |
| 43 | 37309 | 37545 | + | 78 | YP_010078622.1 | hypothetical protein | Ralstonia phage Claudette | 92.31 | 1.22E-44 |
| 44 | 37571 | 38230 | + | 219 | YP_010078621.1 | hypothetical protein | Ralstonia phage Claudette | 97.26 | 5.53E-151 |
| 45 | 38227 | 38442 | + | 71 | QKW95351.1 | hypothetical protein | Ralstonia phage RPZH6 | 91.55 | 2.32E-39 |
| 46 | 38439 | 39212 | + | 257 | no hit | hypothetical protein | no hit |  |  |
| 47 | 39205 | 39717 | + | 170 | YP_010078186.1 | hypothetical protein | Ralstonia phage Raharianne | 69.79 | 1.06E-36 |
| 48 | 40138 | 40335 | + | 65 | YP_010078576.1 | hypothetical protein | Ralstonia phage Firinga | 98.48 | 6.18E-37 |
| 49 | 40311 | 40562 | + | 83 | WAX26289.1 | hypothetical protein | Ralstonia phage p2110 | 98.65 | 1.34E-44 |
| 50 | 40562 | 40660 | + | 32 | YP_010078617.1 | hypothetical protein | Ralstonia phage Claudette | 100.00 | 9.65E-16 |
| 51 | 40660 | 40851 | + | 63 | YP_010078616.1 | hypothetical protein | Ralstonia phage Claudette | 95.24 | 7.45E-37 |
| 52 | 40921 | 42492 | + | 523 | CAH0532131.1 | Phage terminase large subunit | Ralstonia phage UAM5 | 87.95 | 0 |
| 53 | 42489 | 42914 | + | 141 | UAW00964.1 | hypothetical protein | Ralstonia phage RPZH3 | 98.58 | 3.23E-101 |
| 54 | 43139 | 43462 | + | 107 | YP_010078740.1 | HNH endonuclease | Ralstonia phage GP4 | 82.24 | 4.09E-62 |
| 55 | 43462 | 45807 | + | 781 | YP_010078739.1 | portal protein | Ralstonia phage GP4 | 98.59 | 0 |
| 56 | 45818 | 46768 | + | 316 | QKW95340.1 | hypothetical protein | Ralstonia phage RPZH6 | 89.87 | 4.06E-148 |
| 57 | 46782 | 46979 | + | 65 | QKW95339.1 | carbon storage regulator | Ralstonia phage RPZH6 | 93.85 | 5.60E-33 |
| 58 | 47055 | 47348 | + | 97 | YP_010078405.1 | hypothetical protein | Ralstonia phage Gerry | 86.87 | 1.38E-50 |
| 59 | 47408 | 47902 | + | 164 | YP_010078406.1 | hypothetical protein | Ralstonia phage Gerry | 63.64 | 3.79E-61 |
| 60 | 47970 | 49079 | + | 369 | YP_010078609.1 | major head protein | Ralstonia phage Claudette | 99.19 | 4.34E-267 |
| 61 | 49163 | 49630 | + | 155 | UAW00953.1 | virion associated protein | Ralstonia phage RPZH3 | 99.35 | 4.22E-102 |
| 62 | 49683 | 50222 | + | 179 | WAX26301.1 | hypothetical protein | Ralstonia phage p2110 | 98.32 | 1.22E-119 |
| 63 | 50226 | 50888 | + | 220 | YP_010078606.1 | structural protein | Ralstonia phage Claudette | 99.09 | 1.26E-148 |
| 64 | 50885 | 51505 | + | 206 | YP_010078605.1 | virion structural protein | Ralstonia phage Claudette | 99.51 | 1.06E-149 |
| 65 | 51515 | 51937 | + | 140 | YP_010078676.1 | head protein | Ralstonia phage Gervaise | 100.00 | 7.65E-95 |
| 66 | 51944 | 52921 | + | 325 | WAX26305.1 | hypothetical protein | Ralstonia phage p2110 | 99.38 | 8.48E-167 |
| 67 | 52918 | 54624 | + | 568 | QMV32784.1 | hypothetical protein | Ralstonia phage Darius | 98.77 | 4.98e-314 |
| 68 | 54624 | 54905 | + | 93 | WAX26307.1 | hypothetical protein | Ralstonia phage p2110 | 98.92 | 4.81E-60 |
| 69 | 54905 | 56224 | + | 439 | YP_010078808.1 | tail fiber protein Ⅰ | Ralstonia phage GP4 | 97.72 | 0 |
| 70 | 56310 | 57197 | + | 295 | YP_010078807.1 | tail fiber protein Ⅱ | Ralstonia phage GP4 | 98.64 | 3.12E-161 |
| 71 | 57209 | 58942 | + | 577 | QMV32780.1 | head closure protein 3 | Ralstonia phage Darius | 99.83 | 0 |
| 72 | 58942 | 59262 | + | 106 | QKW95408.1 | hypothetical protein | Ralstonia phage RPZH6 | 100.00 | 5.68E-65 |
| 73 | 59338 | 59784 | + | 148 | YP_010078596.1 | acyl-CoA N-acyltransferase | Ralstonia phage Claudette | 100.00 | 1.57E-109 |
| 74 | 59774 | 60607 | + | 277 | YP_010078595.1 | virion structural protein | Ralstonia phage Claudette | 100.00 | 1.24E-181 |
| 75 | 61328 | 60594 | - | 244 | no hit | hypothetical protein | no hit |  |  |

**Table S5** ORF analysis of the YL4 genome.

| ORF | START | END | STRAND | Length(aa) | Accession | Top BLAST hit | Species | identity(%) | Evalue |
| --- | --- | --- | --- | --- | --- | --- | --- | --- | --- |
| 1 | 1 | 1530 | + | 509 | YP_010078593.1 | virion structural protein | Ralstonia phage Claudette | 94.11 | 6.09E-299 |
| 2 | 1619 | 14899 | + | 4426 | YP_010078688.1 | DarB-like antirestriction | Ralstonia phage Gervaise | 92.58 | 0 |
| 3 | 15197 | 14877 | - | 106 | WAX26341.1 | hypothetical protein | Ralstonia phage p2110 | 97.92 | 3.93E-60 |
| 4 | 15361 | 15969 | + | 202 | YP_010078318.1 | endolysin | Ralstonia phage Eline | 96.04 | 1.23E-137 |
| 5 | 15966 | 16193 | + | 75 | YP_010078796.1 | holin | Ralstonia phage GP4 | 100.00 | 7.19E-47 |
| 6 | 16286 | 16666 | + | 126 | YP_010078693.1 | hol-like chemotaxis | Ralstonia phage Gervaise | 100.00 | 2.39E-80 |
| 7 | 16641 | 17045 | + | 134 | YP_010078694.1 | hypothetical protein | Ralstonia phage Gervaise | 100.00 | 2.61E-73 |
| 8 | 17042 | 17578 | + | 178 | YP_010078206.1 | Rz-like spanin | Ralstonia phage Cimandef | 95.51 | 4.35E-108 |
| 9 | 17625 | 17828 | + | 67 | YP_010078792.1 | hypothetical protein | Ralstonia phage GP4 | 98.48 | 1.59E-35 |
| 10 | 19020 | 17818 | - | 400 | YP_010078791.1 | tyrosine recombinase | Ralstonia phage GP4 | 100.00 | 7.28E-293 |
| 11 | 19832 | 19275 | - | 185 | no hit | hypothetical protein | no hit |  |  |
| 12 | 20141 | 19866 | - | 91 | YP_010078698.1 | excisionase | Ralstonia phage Gervaise | 80.68 | 4.14E-46 |
| 13 | 21460 | 20147 | - | 437 | WAX26344.1 | hypothetical protein | Ralstonia phage p2110 | 87.78 | 1.60E-221 |
| 14 | 22553 | 21489 | - | 354 | QKW95389.1 | DNA polymerase III subunit beta | Ralstonia phage RPZH6 | 97.74 | 1.10E-241 |
| 15 | 22805 | 22578 | - | 75 | QKW95387.1 | DNA-binding protein | Ralstonia phage RPZH6 | 100.00 | 1.24E-47 |
| 16 | 23770 | 22811 | - | 319 | CAH0532166.1 | Recombinational DNA repair protein RecT | Ralstonia phage UAM5 | 95.92 | 7.75E-215 |
| 17 | 24783 | 23809 | - | 324 | YP_010078325.1 | RecE-like recombination exonuclease | Ralstonia phage Eline | 97.84 | 6.24E-226 |
| 18 | 25028 | 25504 | + | 158 | CAH0532169.1 | Phage HNH homing endonuclease | Ralstonia phage UAM5 | 88.12 | 2.67E-60 |
| 19 | 26060 | 25506 | - | 184 | YP_010078705.1 | hypothetical protein | Ralstonia phage Gervaise | 93.48 | 3.58E-119 |
| 20 | 26225 | 26593 | + | 122 | QKW95382.1 | transcriptional regulator | Ralstonia phage RPZH6 | 100.00 | 3.87E-75 |
| 21 | 26753 | 27325 | + | 190 | YP_010078648.1 | plasmid-derived single-stranded DNA-binding protein | Ralstonia phage Claudette | 100.00 | 5.95E-130 |
| 22 | 27334 | 27555 | + | 73 | WAX26324.1 | hypothetical protein | Ralstonia phage p2110 | 100.00 | 3.09E-47 |
| 23 | 27569 | 28036 | + | 155 | WAX26325.1 | hypothetical protein | Ralstonia phage p2110 | 95.48 | 1.60E-101 |
| 24 | 28036 | 28416 | + | 126 | WAX26326.1 | hypothetical protein | Ralstonia phage p2110 | 93.55 | 4.76E-85 |
| 25 | 28581 | 29324 | + | 247 | WAX26327.1 | hypothetical protein | Ralstonia phage p2110 | 86.64 | 4.91E-148 |
| 26 | 29321 | 29773 | + | 150 | QKW95374.1 | hypothetical protein | Ralstonia phage RPZH6 | 88.00 | 5.21E-91 |
| 27 | 30034 | 30297 | + | 87 | YP_010078447.1 | hypothetical protein | Ralstonia phage Gerry | 98.72 | 3.71E-46 |
| 28 | 30822 | 31250 | + | 142 | YP_010078767.1 | hypothetical protein | Ralstonia phage GP4 | 99.30 | 3.20E-96 |
| 29 | 31237 | 31893 | + | 218 | WAX26332.1 | hypothetical protein | Ralstonia phage p2110 | 97.71 | 1.36E-142 |
| 30 | 31976 | 32380 | + | 134 | WAX26333.1 | hypothetical protein | Ralstonia phage p2110 | 81.48 | 4.74E-61 |
| 31 | 32377 | 32691 | + | 104 | CAB4154600.1 | hypothetical protein | uncultured Caudovirales phage | 62.90 | 1.10E-20 |
| 32 | 32678 | 33025 | + | 115 | YP_010078718.1 | terminase small subunit | Ralstonia phage Gervaise | 90.60 | 1.25E-64 |
| 33 | 33051 | 33305 | + | 84 | YP_010078225.1 | hypothetical protein | Ralstonia phage Cimandef | 98.81 | 1.30E-53 |
| 34 | 33290 | 33772 | + | 160 | YP_010078226.1 | hypothetical protein | Ralstonia phage Cimandef | 94.19 | 2.48E-102 |
| 35 | 34727 | 34299 | - | 142 | YP_010078277.1 | hypothetical protein | Ralstonia phage Eline | 90.85 | 1.27E-89 |
| 36 | 35085 | 35723 | + | 212 | YP_010078629.1 | terminase small subunit | Ralstonia phage Claudette | 95.28 | 1.86E-137 |
| 37 | 35879 | 36073 | + | 64 | no hit | hypothetical protein | no hit |  |  |
| 38 | 36102 | 36410 | + | 102 | YP_010078755.1 | hypothetical protein | Ralstonia phage GP4 | 84.40 | 4.00E-51 |
| 39 | 36464 | 36622 | + | 52 | YP_010078754.1 | hypothetical protein | Ralstonia phage GP4 | 98.08 | 1.01E-23 |
| 40 | 36619 | 36762 | + | 47 | YP_010078753.1 | hypothetical protein | Ralstonia phage GP4 | 97.87 | 1.92E-21 |
| 41 | 36759 | 37109 | + | 116 | YP_008853822.1 | hypothetical protein | Ralstonia phage RSK1 | 94.83 | 1.62E-71 |
| 42 | 37106 | 37342 | + | 78 | YP_010078622.1 | hypothetical protein | Ralstonia phage Claudette | 92.31 | 1.22E-44 |
| 43 | 37368 | 38027 | + | 219 | YP_010078621.1 | hypothetical protein | Ralstonia phage Claudette | 97.26 | 5.53E-151 |
| 44 | 38024 | 38239 | + | 71 | QKW95351.1 | hypothetical protein | Ralstonia phage RPZH6 | 91.55 | 2.32E-39 |
| 45 | 38236 | 39075 | + | 279 | no hit | hypothetical protein | no hit |  |  |
| 46 | 39135 | 39332 | + | 65 | YP_009792735.1 | hypothetical protein | Bordetella phage vB_BbrM_PHB04 | 66.04 | 4.07E-20 |
| 47 | 39496 | 39693 | + | 65 | YP_010078576.1 | hypothetical protein | Ralstonia phage Firinga | 98.48 | 6.18E-37 |
| 48 | 39669 | 39920 | + | 83 | WAX26289.1 | hypothetical protein | Ralstonia phage p2110 | 98.65 | 1.34E-44 |
| 49 | 39920 | 40018 | + | 32 | YP_010078617.1 | hypothetical protein | Ralstonia phage Claudette | 100.00 | 9.65E-16 |
| 50 | 40018 | 40209 | + | 63 | YP_010078616.1 | hypothetical protein | Ralstonia phage Claudette | 95.24 | 7.45E-37 |
| 51 | 40279 | 41850 | + | 523 | CAH0532131.1 | Phage terminase large subunit | Ralstonia phage UAM5 | 87.95 | 0 |
| 52 | 41847 | 42272 | + | 141 | UAW00964.1 | hypothetical protein | Ralstonia phage RPZH3 | 98.58 | 3.23E-101 |
| 53 | 42497 | 42820 | + | 107 | YP_010078740.1 | HNH endonuclease | Ralstonia phage GP4 | 82.24 | 4.09E-62 |
| 54 | 42820 | 45165 | + | 781 | YP_010078667.1 | portal protein | Ralstonia phage Gervaise | 99.49 | 0 |
| 55 | 45176 | 46126 | + | 316 | QKW95340.1 | hypothetical protein | Ralstonia phage RPZH6 | 90.19 | 2.86E-148 |
| 56 | 46152 | 46412 | + | 86 | YP_010078737.1 | hypothetical protein | Ralstonia phage GP4 | 97.67 | 5.62E-59 |
| 57 | 46439 | 46636 | + | 65 | YP_010078299.1 | CsrA-like regulator | Ralstonia phage Eline | 92.31 | 5.60E-33 |
| 58 | 0.46893 | 46633 | - | 86 | YP_010078300.1 | HNH endonuclease | Ralstonia phage Eline | 80.23 | 1.22E-42 |
| 59 | 47319 | 48428 | + | 369 | YP_010078248.1 | major head protein | Ralstonia phage Cimandef | 98.92 | 7.50E-268 |
| 60 | 48512 | 48979 | + | 155 | YP_010078249.1 | virion structural protein | Ralstonia phage Cimandef | 98.71 | 1.47E-102 |
| 61 | 49032 | 49571 | + | 179 | YP_010078303.1 | hypothetical protein | Ralstonia phage Eline | 97.77 | 1.22E-119 |
| 62 | 49575 | 50237 | + | 220 | YP_010078814.1 | structural protein | Ralstonia phage GP4 | 97.73 | 1.21E-146 |
| 63 | 50234 | 50854 | + | 206 | WAX26303.1 | hypothetical protein | Ralstonia phage p2110 | 100.00 | 1.02E-147 |
| 64 | 50864 | 51286 | + | 140 | WAX26304.1 | hypothetical protein | Ralstonia phage p2110 | 100.00 | 1.09E-94 |
| 65 | 51293 | 52270 | + | 325 | WAX26305.1 | hypothetical protein | Ralstonia phage p2110 | 98.15 | 5.40E-163 |
| 66 | 52267 | 53973 | + | 568 | WAX26306.1 | hypothetical protein | Ralstonia phage p2110 | 99.47 | 2.87e-313 |
| 67 | 53973 | 54254 | + | 93 | WAX26307.1 | hypothetical protein | Ralstonia phage p2110 | 98.92 | 4.81E-60 |
| 68 | 54254 | 55573 | + | 439 | YP_010078808.1 | tail fiber protein Ⅰ | Ralstonia phage GP4 | 97.72 | 0 |
| 69 | 55659 | 56531 | + | 290 | WAX26309.1 | tail fiber protein Ⅱ | Ralstonia phage p2110 | 100.00 | 7.83E-164 |
| 70 | 56543 | 58276 | + | 577 | QMV32780.1 | head closure protein 3 | Ralstonia phage Darius | 98.44 | 0 |
| 71 | 58276 | 58596 | + | 106 | YP_010078597.1 | hypothetical protein | Ralstonia phage Claudette | 99.06 | 2.31E-64 |
| 72 | 58672 | 59118 | + | 148 | YP_010078596.1 | acyl-CoA N-acyltransferase | Ralstonia phage Claudette | 100.00 | 1.57E-109 |
| 73 | 59108 | 59941 | + | 277 | YP_010078595.1 | virion structural protein | Ralstonia phage Claudette | 98.92 | 5.91E-180 |
| 74 | 60662 | 59928 | - | 244 | no hit | hypothetical protein | no hit |  |  |
